# Inhibition of ERK1/2 or CRMP2 Disrupts Alcohol Memory Reconsolidation and Prevents Relapse in Rats

**DOI:** 10.3390/ijms25105478

**Published:** 2024-05-17

**Authors:** Nofar Rahamim, Mirit Liran, Coral Aronovici, Hila Flumin, Tamar Gordon, Nataly Urshansky, Segev Barak

**Affiliations:** 1 Sagol School of Neuroscience, Tel Aviv University, Tel Aviv 69978, Israel; nofarrahamim@mail.tau.ac.il (N.R.);; 2 School of Psychological Sciences, Tel Aviv University, Tel Aviv 69978, Israelnaturshan@gmail.com (N.U.); 3 Faculty of Life Sciences, Department of Neurobiology, Tel Aviv University, Tel Aviv 69978, Israel

**Keywords:** alcohol, addiction, memory reconsolidation, signaling, ERK1/2, CRMP2

## Abstract

Relapse to alcohol abuse, often caused by cue-induced alcohol craving, is a major challenge in alcohol addiction treatment. Therefore, disrupting the cue-alcohol memories can suppress relapse. Upon retrieval, memories transiently destabilize before they reconsolidate in a process that requires protein synthesis. Evidence suggests that the mammalian target of rapamycin complex 1 (mTORC1), governing the translation of a subset of dendritic proteins, is crucial for memory reconsolidation. Here, we explored the involvement of two regulatory pathways of mTORC1, phosphoinositide 3-kinase (PI3K)-AKT and extracellular regulated kinase 1/2 (ERK1/2), in the reconsolidation process in a rat (Wistar) model of alcohol self-administration. We found that retrieval of alcohol memories using an odor-taste cue increased ERK1/2 activation in the amygdala, while the PI3K-AKT pathway remained unaffected. Importantly, ERK1/2 inhibition after alcohol memory retrieval impaired alcohol-memory reconsolidation and led to long-lasting relapse suppression. Attenuation of relapse was also induced by post-retrieval administration of lacosamide, an inhibitor of collapsin response mediator protein-2 (CRMP2)—a translational product of mTORC1. Together, our findings indicate the crucial role of ERK1/2 and CRMP2 in the reconsolidation of alcohol memories, with their inhibition as potential treatment targets for relapse prevention.

## 1. Introduction

Relapse prevention is a significant challenge in treating AUD [1,2], and is often driven by cue-induced cravings, caused by re-exposure to alcohol-associated cues [3,4,5,6,7]. Thus, disruption of the memories for the cue-alcohol association is expected to suppress cue-induced relapse.

It is believed that upon their retrieval, memories become temporarily unstable during an active state, and then undergo a process of re-stabilization, termed memory reconsolidation. Memories have been shown to be labile during their reconsolidation process and consequently can be manipulated [8,9,10,11]. The therapeutic potential of interference with the reconsolidation process in attenuating or altering maladaptive associative memories has been increasingly acknowledged [5,12,13,14,15,16,17]. Thus, it has been demonstrated that the inhibition of protein synthesis during memory reconsolidation (i.e., following memory retrieval) suppresses the behavioral expression of various types of memories [18,19,20,21,22,23,24], including prevention of alcohol relapse [19,23], suggesting that memory reconsolidation requires de-novo protein synthesis [8,18,25].

We previously demonstrated that the mammalian target of rapamycin complex 1 (mTORC1), essential for translating dendritic proteins and involved in synaptic plasticity and memory [26,27,28], plays a crucial role in alcohol memory reconsolidation [19]. Specifically, alcohol memory retrieval in rats with a history of long-term alcohol intake activated mTORC1 signaling in the central amygdala (CeA), the medial prefrontal cortex (mPFC), and the orbitofrontal cortex (OFC), enhancing the expression of several mTORC1-regulated synaptic proteins [19]. The administration of mTORC1 inhibitor rapamycin following alcohol memory retrieval suppressed relapse without affecting sucrose memories [19]. Further research showed that post-retrieval rapamycin also reduced alcohol-, morphine-, and cocaine-conditioned place preference [23].

The canonical mTORC1 activator is the phosphoinositide 3-kinase (PI3K)-AKT signaling pathway [29,30,31,32,33]. When active, AKT phosphorylates, and thereby inactivates, the glycogen synthase kinase 3β (GSK3β) [34]. Activation of AKT-GSK3β-mTORC1 signaling was observed during the reconsolidation of cocaine [35,36,37,38] and heroin memories [39]. Moreover, inhibition of PI3K [40] or AKT-dependent activation of mTORC1 [41] was shown to impair fear memory reconsolidation, further implying on the involvement of PI3K-AKT-mTORC1 signaling in the reconsolidation process.

Another positive regulator of mTORC1 is the extracellular regulated kinase 1/2 (ERK1/2), which is part of the mitogen-activated protein kinase (MAPK) signaling pathway [29,30,42]. Cue-induced relapse to alcohol-seeking [43,44] was shown to increase ERK1/2 signaling, whereas inhibition of ERK1/2 impaired the reconsolidation of cocaine and morphine memories [45,46,47,48]. This suggests that ERK1/2 activation plays a role in the reconsolidation of drug-associated memories.

Both the PI3K-AKT and the ERK1/2 signaling pathways were shown to be involved in alcohol consumption and relapse. Specifically, PI3K-AKT signaling is thought to underlie the mTORC1 activity that contributes to the escalation and maintenance of alcohol-drinking phenotypes [29,30,31,32,33]. In addition, the GSK3β substrate and mTORC1 downstream product, collapsin response mediator protein-2 (CRMP2) [49], which promotes microtubule assembly and neurite outgrowth [50,51,52], was implicated in relapse to alcohol seeking [53]. Conversely, ERK1/2 signaling has primarily been characterized as a mechanism that gates the level of alcohol intake [31,54,55,56]. Nonetheless, besides mTORC1, the role of these signaling pathways in the reconsolidation of alcohol memories has not been directly investigated.

Here, we characterized the involvement of the PI3K-AKT and ERK1/2 signaling pathways in alcohol memory reconsolidation. To this end, we used the odor-taste cue retrieval method, previously shown to elicit mTORC1 signaling [19]. We examined the effects of alcohol memory retrieval on the activation of several effectors of these pathways, focusing on the mPFC, the whole amygdala and nucleus accumbens (NAc), brain regions previously implicated in the reconsolidation of drug memories [19,35,37,47,48,57,58]. To establish causality, we tested whether inhibition of certain elements in these signaling pathways, namely CRMP2, GSK3β, and ERK1/2, impair the reconsolidation process and prevent relapse to alcohol drinking.

## 2. Results

### 2.1. Alcohol Memory Retrieval Does Not Lead to Activation of the PI3K-AKT Signaling Pathway

**Figure 1:**
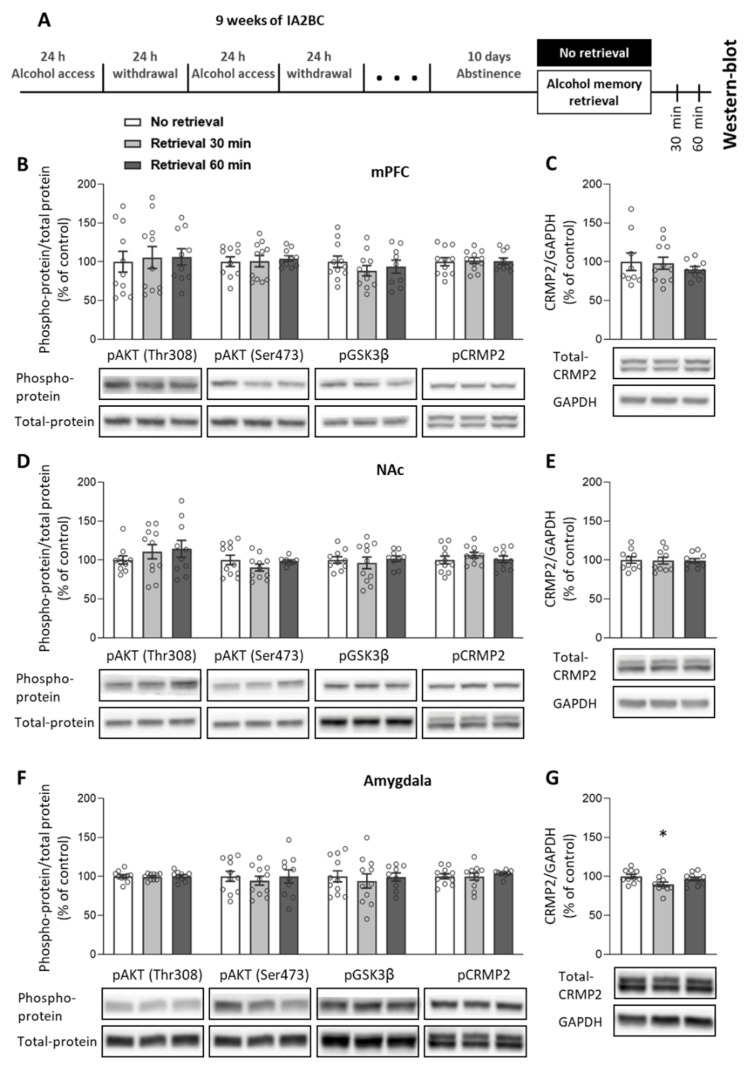
Alcohol memory retrieval does not affect the activation of the PI3K-AKT signaling pathway. (**A**) Experimental design and timeline. Rats consumed alcohol in the intermittent access to 20% alcohol in a 2-bottle choice paradigm for nine weeks, followed by 10 days of abstinence. After the abstinence period, alcohol memory was retrieved using an odor-taste cue. Tissues were collected 30 min and 60 min after the retrieval. Phosphorylation and total protein levels in the medial prefrontal cortex (mPFC), nucleus accumbens (NAc) and whole amygdala were determined by western blot. (**B**–**G**) Phospho-protein levels of AKT (Thr308, Ser473), glycogen synthase kinase 3β (GSK3β) (Ser9), and collapsin response mediator protein-2 (CRMP2) (Thr514) were normalized to the total protein immunoreactivity (**B**,**D**,**F**). Total protein levels of CRMP2 were normalized to glyceraldehyde 3-phosphate dehydrogenase (GAPDH) (**C**,**E**,**G**). Bar graphs represent mean ± SEM of the percent of change from the control No retrieval group. *n* = 9–11 (4–5 males, 4–6 females). * *p* < 0.05 (No retrieval vs. Retrieval 30 min).

As we previously found that alcohol memory retrieval activates the mTORC1 pathway [19], we examined whether the retrieval of alcohol memories activates the AKT-PI3K signaling pathway, upstream of mTORC1. Rats were trained to consume alcohol through the intermittent access to 20% alcohol in 2-bottle choice (IA2BC) procedure for nine weeks. The average alcohol intake during the last five drinking sessions was 4.22 g/kg (SD = 1.65). Rats were then subjected to 10 days of abstinence. On the 11th day of abstinence, alcohol memories were retrieved via the presentation of an odor-taste cue (see Section 4). We then determined the phosphorylation levels of AKT, GSK3β, and CRMP2 in the mPFC, NAc, and the whole amygdala, 30 and 60 min after memory retrieval. Since CRMP2 is also a translational product of mTORC1 activation [59], we also examined changes in the total protein levels of CRMP2 (Figure 1A; Experiment 1 in Section 4).

As shown in Figure 1B,D,F, retrieval of alcohol memory did not affect the phosphorylation levels of AKT, GSK3β, and CRMP2 (one-way ANOVA: mPFC: pAKT (Thr308) [F(2,29) = 0.07, *p* = 0.935), pAKT (Ser473) [F(2,28) = 0.14, *p* = 0.874], pGSK3β [F(2,28) = 0.66, *p* = 0.523], pCRMP2 [F(2,29) = 0.03, *p* = 0.975]; NAc: pAKT (Thr308) [F(2,28) = 0.68, *p* = 0.514], pAKT (Ser473) [F(2,28) = 1.28, *p* = 0.293], pGSK3β [F(2,27) = 0.24, *p* = 0.791], pCRMP2: [F(2,28) = 0.6, *p* = 0.558]; Amygdala: pAKT (Thr308) [F(2,27) = 0.04, *p* = 0.961], pAKT (Ser473) [F(2,29) = 0.23, *p* = 0.793], pGSK3β [F(2,28) = 0.19, *p* = 0.831], pCRMP2 [F(2,28) = 0.39, *p* = 0.684]) (Figures S1–S3).

The levels of the total CRMP2 protein were mostly unaffected as well, except for a mild reduction observed in the amygdala 30 min after alcohol memory retrieval (Figure 1C,E,G and Figures S1–S3; one-way ANOVA: Amygdala, F(2,27) = 3.67, *p* = 0.038. Dunnett post hoc: No-retrieval vs. retrieval 30-min, *p* = 0.024; mPFC [F(2,26) = 0.39, *p* = 0.679]; NAc [F(2,27) = 0.02, *p* = 0.982]).

Since activation of mTORC1 was previously observed in the OFC following exposure to a context that was associated with alcohol self-administration, we also investigated this region. However, no changes in the inspected proteins were observed (Figure S4).

These findings suggest that the retrieval of alcohol memories and their reconsolidation do not activate PI3K-AKT-GSK3β signaling.

### 2.2. CRMP2 Inhibition Following Alcohol Memory Retrieval Reduces Subsequent Alcohol Consumption

We found no CRMP2 activation following alcohol memory retrieval. However, it was previously reported that prime-induced reinstatement of alcohol-seeking increased the total protein levels of CRMP2, and that inhibition of this protein reduced reinstatement [53]. Therefore, we next investigated whether inhibition of CRMP2 would affect alcohol memory reconsolidation, resulting in reduced relapse to alcohol drinking. To this end, we used lacosamide, an FDA-approved antiepileptic drug [60], which inhibits CRMP2’s ability to promote tubulin polymerization, and thereby impairs neurite outgrowth and branching [61,62].

**Figure 2:**
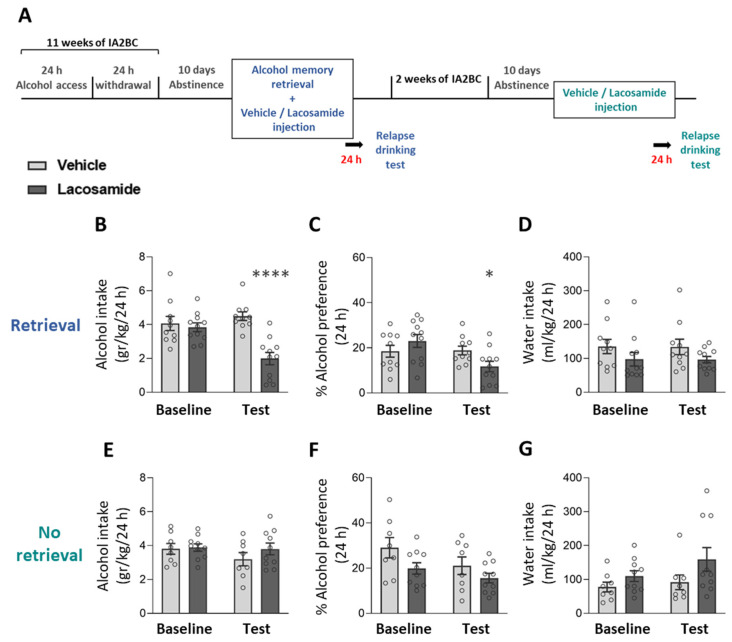
Lacosamide treatment after alcohol memory retrieval suppresses relapse to alcohol consumption. (**A**) Experimental design and timeline. Rats consumed alcohol in the intermittent access to 20% alcohol 2-bottle choice paradigm for 11 weeks. Following 10 days of abstinence, alcohol memory was retrieved using an odor-taste cue. Immediately afterward, rats were injected with lacosamide (20 mg/kg) or vehicle. Relapse to alcohol drinking was tested the next day, in a 24-h 2-bottle choice drinking session. After a 2-week re-training, the rats were subjected again to 10 days of abstinence and were then given an additional treatment of lacosamide or vehicle, without a preceding alcohol memory retrieval, and relapse was tested 24 h later. (**B**–**G**) Alcohol intake (**B**,**E**), alcohol preference (**C**,**F**), and water intake (**D**,**G**) in a baseline and relapse test conducted a day after lacosamide treatment given following alcohol memory retrieval (**B**–**D**) or without memory retrieval (**E**–**G**). Baseline levels of alcohol/water intake/preference represent the average of the last five (**B**–**D**) or three sessions (**E**–**G**) prior to abstinence. Bar graphs represent mean ± SEM. *n* = 8–11 (males: 4–6, females: 3–6) per group. * *p* < 0.05, **** *p* < 0.0001 (Vehicle vs. Lacosamide).

Rats were trained to consume alcohol in the IA2BC procedure for 11 weeks and were then subjected to 10 days of abstinence. On the next day, alcohol memory was retrieved, and rats were injected with lacosamide (20 mg/kg) or vehicle immediately afterward. Relapse to alcohol drinking was tested 24 h later (Figure 2A; Experiment 2 in Section 4).

As shown in Figure 2B–D, rats treated with lacosamide following memory retrieval consumed less alcohol and showed a lower preference for alcohol compared with vehicle-treated controls, without affecting water intake (*t*-test: alcohol intake [t(19) = 5.50, *p* < 0.0001]; alcohol preference [t(19) = 2.31, *p* = 0.032]; water intake [t(19) = 1.58, *p* = 0.131]).

To validate that the effect of lacosamide on relapse to alcohol drinking was due to an impaired reconsolidation process, i.e., that alcohol memory retrieval is required prior to lacosamide injection for the effect to occur, we tested the effects of lacosamide without prior memory retrieval. To this end, we re-trained the rats in the IA2BC for two additional weeks. We then repeated the experiment as above, except that lacosamide was injected without prior memory retrieval, and a relapse to alcohol drinking test was held on the next day.

As shown in Figure 2E–G, lacosamide treatment without prior alcohol memory retrieval had no effects on the rats’ relapse to alcohol drinking or preference (*t*-test: alcohol intake [t(16) = 1.16, *p* = 0.263]; alcohol preference [t(16) = 1.3, *p* = 0.213]), or water intake (t(16) = 1.55, *p* = 0.141]).

These results suggest that inhibition of CRMP2 after alcohol memory retrieval impairs the reconsolidation of alcohol memories, and thereby attenuates post-abstinence relapse to alcohol consumption.

### 2.3. Post-Retrieval GSK3β Inhibition Does Not Affect Relapse to Alcohol Consumption

The ability of CRMP2 to bind to tubulin is negatively regulated by GSK3β [49,63]. Moreover, GSK3β inhibition was previously shown to disrupt the reconsolidation of heroin [39] and cocaine [35,36,37,38] memories. Since we found that administration of the CRMP2 inhibitor, lacosamide, impairs the reconsolidation of alcohol memory, we next examined whether the inhibition of GSK3β following alcohol memory retrieval will also affect alcohol memory reconsolidation and relapse.

**Figure 3:**
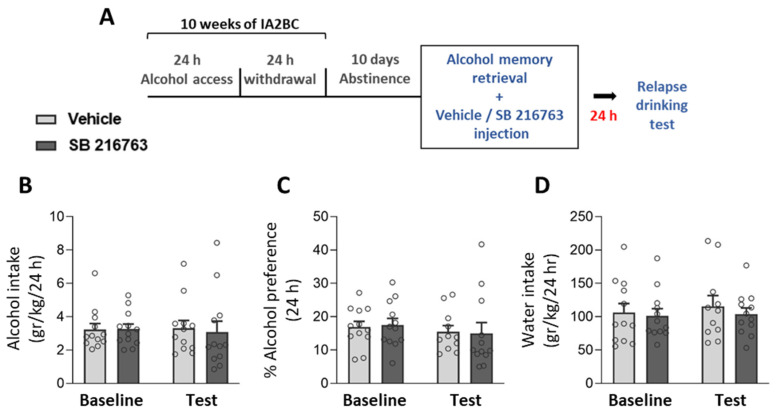
GSK3β inhibition following alcohol memory retrieval does not affect relapse to alcohol consumption. (**A**) Experimental design and timeline. Rats consumed alcohol in the intermittent access to 20% alcohol in a 2-bottle choice paradigm for 10 weeks, followed by 10 days of abstinence. After the abstinence period, alcohol memory was retrieved using an odor-taste cue. Immediately afterward, rats were injected with SB 216763 (2.5 mg/kg) or vehicle. Relapse to alcohol drinking was tested the next day, in a 24-h 2-bottle choice drinking session. (**B**,**C**) Alcohol intake (**B**), alcohol preference (**C**), and water intake (**D**) in a baseline and relapse test conducted a day after the SB 216763 treatment. Baseline levels of alcohol intake/preference represent the average of the last five sessions prior to abstinence. Bar graphs represent mean ± SEM. *n* = 11–12 (males: 6, females: 5–6) per group.

Rats consumed alcohol as described above. Following 10 days of abstinence, they were injected with the GSK3β inhibitor SB 216763 (2.5 mg/kg) or vehicle immediately after memory retrieval. A relapse test was conducted 24 h later (Figure 3A; Experiment 3 in Section 4).

We found that SB 216763 had no effects on alcohol or water consumption or alcohol preference (Figure 3B–D; *t*-tests: alcohol intake [t(22) = 0.29, *p* = 0.775]; alcohol preference [t(21) = 0.12, *p* = 0.902]; water intake [t(21) = 0.66, *p* = 0.514]). These results indicate that inhibition of GSK3β does not affect alcohol memory reconsolidation.

### 2.4. Alcohol Memory Retrieval Increases ERK1/2 Activation in the Amygdala

As we found no indication for activation (phosphorylation) of AKT following alcohol memory retrieval in any of the tested brain regions, we next set out to explore whether ERK1/2, another major signaling cascade that also serves as a positive regulator of mTORC1 [30], is involved in the alcohol memory reconsolidation process.

**Figure 4:**
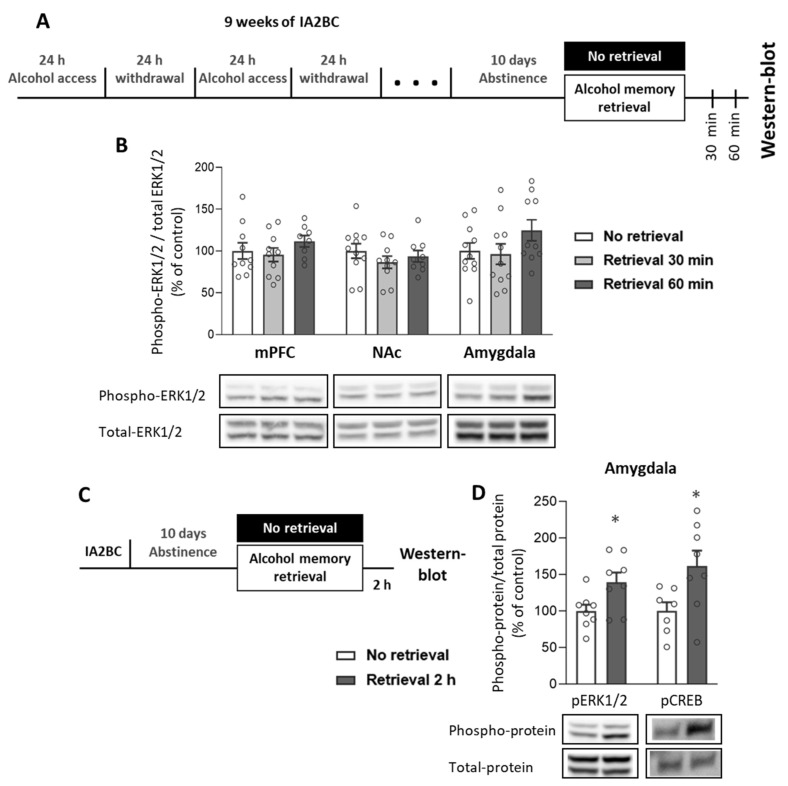
Alcohol memory retrieval increases ERK1/2 and CREB phosphorylation in the amygdala. (**A**) Experimental design and timeline. Rats consumed alcohol in the intermittent access to 20% alcohol in a 2-bottle choice paradigm for nine weeks, followed by 10 days of abstinence. After the abstinence period, alcohol memory was retrieved using an odor-taste cue. Tissues were collected 30 and 60 min following memory retrieval. Phosphorylation and total protein levels were determined by western blot. Phospho-protein levels of extracellular regulated kinase1/2 (ERK1/2) (Thr202, Tyr204) were normalized to the total protein levels. (**B**) Phospho-ERK1/2 levels in the mPFC, NAc, and amygdala. (**C**,**D**) A follow-up experiment, in which tissues were collected two hours after the alcohol memory retrieval ((**C**), experimental design and timeline). (**D**) Phospho-protein levels of ERK1/2 and cAMP response element binding protein (CREB) (Ser133) were normalized to total-protein levels. Bar graphs represent mean ± SEM of the percent of change from the No retrieval control group. *n* = 7–11 (males: 4–5, females: 3–6) per group; * *p* < 0.05.

First, we tested whether the retrieval of alcohol memories affects ERK1/2 phosphorylation, i.e., its activation. We tested samples from the mPFC, NAc, OFC, and whole amygdala, which were collected 30 min and 60 min after the alcohol memory retrieval (Figure 4A; Experiment 1 in Section 4).

We found that alcohol memory retrieval had no significant effects on ERK1/2 phosphorylation 30 min or 60 min after the memory retrieval (Figure 4B and Figures S1–S4 (OFC); one-way ANOVAs: mPFC [F(2,25) = 0.88, *p* = 0.429]; NAc [F(2,27) = 0.79, *p* = 0.464]; amygdala [F(2,29) = 1.75, *p* = 0.192]).

Further examination of ERK1 and ERK2 phosphorylation separately revealed increased phosphorylation of ERK1 and a similar trend in ERK2 in the amygdala 60 min after alcohol memory retrieval (Figure S5). Therefore, we next tested for changes in ERK1/2 phosphorylation in the amygdala two hours after memory retrieval (Figure 4C; Experiment 4 in Section 4). We found a significant increase in the phosphorylation level of ERK1/2 in the group that underwent retrieval of alcohol memory, compared to no retrieval controls (Figure 4D and Figure S6; *t*-test: t(14) = 2.51, *p* = 0.025).

Since ERK1/2 is a positive regulator of cAMP response element binding protein (CREB), which we and others have implicated in memory reconsolidation [47,64,65,66], we next assessed CREB phosphorylation in the amygdala two hours after the memory retrieval. We found that alcohol memory retrieval increased phospho-CREB levels compared with no retrieval controls (Figure 4D and Figure S6; *t*-test: t(13) = 2.46, *p* = 0.029). These findings indicate that amygdala ERK1/2-CREB signaling occurs following alcohol memory retrieval, suggesting that this pathway may be involved in the reconsolidation of alcohol memories.

### 2.5. ERK1/2 Inhibition Following Alcohol Memory Retrieval Reduces Relapse to Alcohol Consumption

Having shown that alcohol memory retrieval increased the phosphorylation (i.e., activation) of ERK1/2 in the amygdala, we next set out to establish the causal role of ERK1/2 signaling in the reconsolidation of alcohol memories. To this end, we next tested the effect of post-retrieval ERK1/2 inhibition on relapse to alcohol drinking. We used SL-327 [67], an inhibitor of mitogen-activated protein kinase 1/2 (MEK1/2), the upstream regulator of ERK1/2.

**Figure 5:**
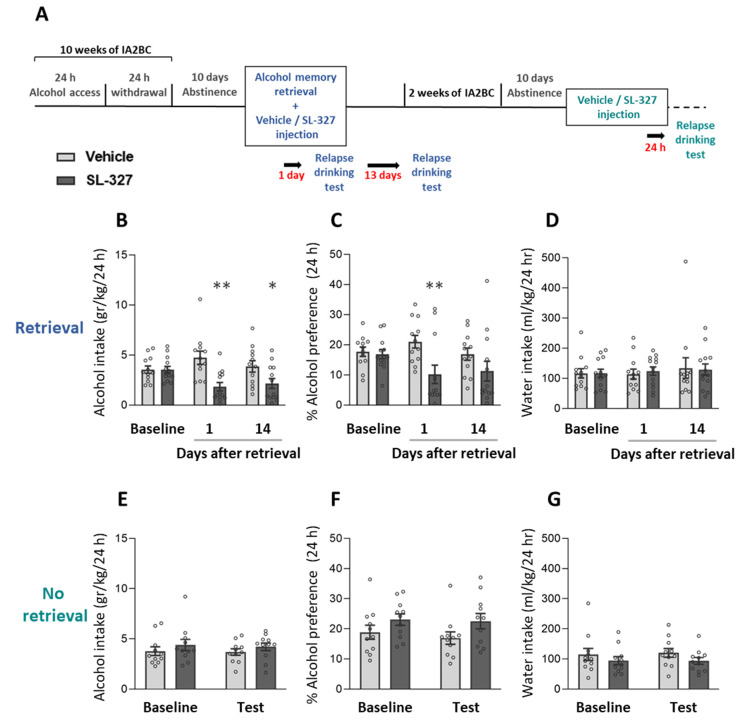
SL-327 treatment after alcohol memory retrieval suppresses relapse to alcohol consumption and preference. (**A**) Experimental design and timeline. Rats consumed alcohol in the intermittent access to 20% alcohol in a 2-bottle choice paradigm for 10 weeks, followed by 10 days of abstinence. After the abstinence period, alcohol memory was retrieved using an odor-taste cue. Immediately afterward, rats were injected with SL-327 (50 mg/kg) or vehicle. Relapse to alcohol drinking was tested 1 and 14 days later in a 24-h 2-bottle choice drinking session. After a 3-week re-training, rats were subjected to 10 days of abstinence and were then given an additional treatment of SL-327 or vehicle, without alcohol memory retrieval, and relapse was tested a day later. (**B**–**G**) Alcohol intake (**B**,**E**), alcohol preference (**C**,**F**), and water intake (**D**,**G**) in a baseline and relapse test conducted after SL-327 treatment given following alcohol memory retrieval (**B**–**D**) or without memory retrieval (**E**–**G**). Baseline levels of alcohol/water intake/preference represent the average of the last five (**B**–**D**) or three (**E**–**G**) sessions prior to abstinence. Bar graphs represent mean ± SEM. *n* = 11–13 (males: 6, females: 6–7) per group. * *p* < 0.05, ** *p* < 0.01 (vehicle vs. SL-327).

After 10 weeks of alcohol consumption and 10 days of abstinence, alcohol memories were retrieved as described above, and immediately afterward the rats were injected with SL-327 (50 mg/kg) or vehicle. Relapse to alcohol drinking was tested 1 and 14 days later. The rats were abstinent from alcohol between the two tests (Figure 5A; Experiment 4 in Section 4).

We found that SL-327-treated rats consumed less alcohol one day and 14 days after the retrieval + SL-327 treatment, compared with vehicle-treated controls. The rats showed lower preference for alcohol one day, but not 14 days, after the treatment (Figure 5B,C; *t*-test: post-retrieval test—1 day: alcohol intake [t(23) = 3.76, *p* = 0.001], alcohol preference [t(23) = 2.87, *p* = 0.009]; post-retrieval test—14 days: alcohol intake [t(23) = 2.28, *p* = 0.033], alcohol preference [t(23) = 1.43, *p* = 0.165]). Water intake was not affected by the treatment (Figure 5D; *t*-test: post-retrieval test—1 day [t(23) = 0.46, *p* = 0.650]; post-retrieval test—14 days [t(23) = 0.14, *p* = 0.893]).

To assess whether SL-327′s effects on relapse to alcohol drinking resulted from impaired reconsolidation, we re-tested the effects of ERK1/2 inhibition without prior memory retrieval. Rats received drinking re-training for three additional weeks, followed by 10 days of abstinence, and were then treated with SL-327 or vehicle in a counterbalanced manner, without prior memory retrieval. A day later, we tested relapse to alcohol drinking.

We found that when given without prior memory retrieval, SL-327 had no effects on the rats’ alcohol or water drinking or on alcohol preference (Figure 5E–G; *t*-tests: alcohol intake [t(20) = 1.05, *p* = 0.305]; alcohol preference [t(20) = 1.72, *p* = 0.101]; water intake [t(20) = 1.46, *p* = 0.161]).

Together, these results indicate that inhibition of ERK1/2 reduces relapse to alcohol consumption in a long-lasting manner, but only when applied following alcohol memory retrieval, suggesting that inhibition of ERK1/2 impairs alcohol memory reconsolidation.

Finally, we validated that the systemic SL-327 injection indeed inhibits ERK1/2 phosphorylation in the amygdala, where we observed increased ERK1/2 phosphorylation following alcohol memory retrieval. In addition to ERK1/2, we also examined changes in the phosphorylation level of S6 ribosomal protein, a downstream effector of mTORC1, which was shown to be elevated in the CeA following alcohol memory retrieval [19] (Experiment 5 in Section 4). We dissected whole amygdala tissues 90 min [68,69] after the systemic injection of SL-327 and processed them for a Western blot analysis.

**Figure 6:**
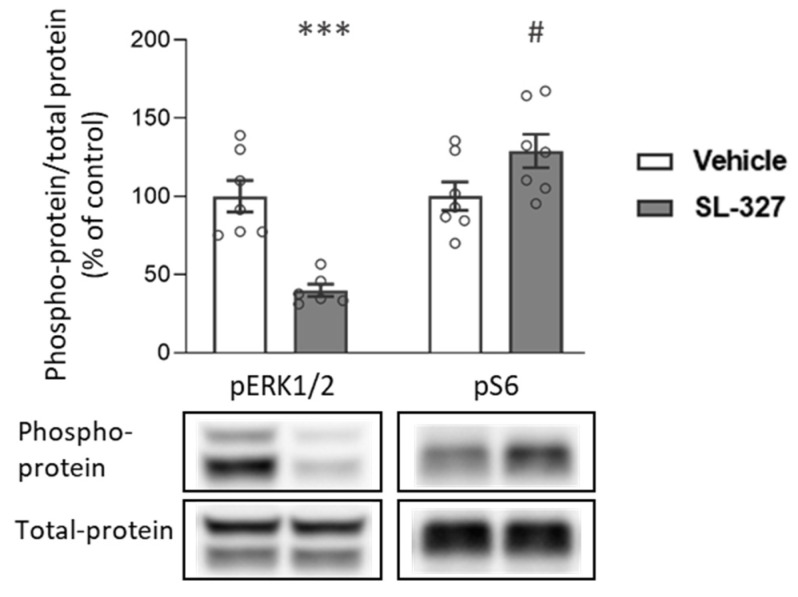
SL-327 injection reduces ERK1/2 phosphorylation in the amygdala. Rats were injected with SL-327 (50 mg/kg) or vehicle. Amygdala tissues were collected 90 min after the injection. Phosphorylation and total protein levels were determined by western blot. Phospho-protein levels of ERK1/2 (Thr202, Tyr204) and S6 (Ser235/236) were normalized to the total-protein immunoreactivity. Bar graphs represent mean ± SEM of the percent of change from the control group. *n* = 6–7 (males: 2–3, females: 4) per group; # *p* = 0.06, *** *p* < 0.001.

As expected, SL-327 treatment led to reduced phosphorylation of ERK1/2 (*t*-test: t(11) = 5.24, *p* = 0.0003). Surprisingly, a trend toward increased phosphorylation of S6 was observed following the SL-327 treatment (*t*-test: t(12) = 2.06, *p* = 0.06) (Figure 6 and Figure S7).

These results imply that the observed effects of ERK1/2 inhibition on alcohol memory reconsolidation and relapse are not necessarily mediated by its effects on mTORC1 activity.

## 3. Discussion

We show here that the retrieval of alcohol-associated memories in rats with a history of long-term alcohol drinking evokes the activation of ERK1/2 signaling specifically in the amygdala while the PI3K-AKT signaling pathway remains inactive throughout the mesocorticolimbic system. Furthermore, we demonstrate that the post-retrieval inhibition of the ERK1/2 pathway results in long-lasting suppression of relapse to alcohol drinking, suggesting that this signaling pathway plays a role in alcohol memory reconsolidation. In addition, we show that relapse is suppressed by the post-retrieval inhibition of CRMP2, a translational product of mTORC1 signaling. In contrast, the post-retrieval inhibition of GSK3β, an effector of the PI3K-AKT signaling pathway that regulates CRMP2 activity [63], does not affect relapse to alcohol consumption. Together, our findings suggest that the ERK1/2 signaling pathway, as well as CRMP2, plays a critical role in alcohol memory reconsolidation, marking them as potential targets for relapse prevention.

### 3.1. ERK1/2 Is Crucial for Alcohol Memory Reconsolidation

Our results suggest that ERK1/2 has a crucial role in the reconsolidation of alcohol memories. These findings are in line with previous reports, showing that cue-induced reinstatement of alcohol seeking (measured by operant response) led to increased ERK1/2 activation in the basolateral amygdala (BLA) [43,44] and the NAc shell [43]. Here, we show that the mere exposure to the drug-associated cues is sufficient to induce ERK1/2 activation, even in the absence of the operant seeking behavior. Of note, since we observed increased ERK1 activation 60 min after the retrieval (Figure S5), we also examined ERK1/2 phosphorylation 2 h after alcohol memory retrieval and found a significantly increased pERK1/2 at this time point. As we had no indications for altered phosphorylation of AKT, GSK3β, or CRMP2 30 or 60 min following the retrieval, they were not further examined at the 2 h time point. Nonetheless, we cannot exclude the possibility that a longer post-retrieval period may induce additional signaling alterations.

Importantly, we found increased ERK1/2 activation in the amygdala, but not in the mPFC, NAc, or OFC, which are also known to be key regions for drug memory reconsolidation [16,19,35,46,58,70,71,72,73,74]. These results extend our previous findings of increased mTORC1 activation following alcohol memory retrieval, using two different retrieval methods [19]. Thus, when the memory was retrieved by exposure to the context of the alcohol self-administration chamber, mTORC1 activation was increased in the CeA and the prelimbic and orbitofrontal cortices. However, when the retrieval was conducted as in our present study, i.e., by the presentation of odor-taste cues in the home cage, mTORC1 activation was increased only in the CeA [19]. Taken together, these findings align with the view that the amygdala encodes the Pavlovian association between the conditioned stimuli (CS; the odor and taste of alcohol) and the unconditioned stimulus (US; the reinforcing effect of alcohol), whereas cortical regions, as well as the NAc, encodes the instrumental and motivational aspects of the memory [6,19,58].

We show that ERK1/2 inhibition following alcohol memory retrieval interferes with the reconsolidation of the alcohol memory, leading to reduced relapse to alcohol drinking in the home cage. In contrast, ERK1/2 inhibition was previously shown to increase alcohol-reinforced operant responding [54,56] and alcohol drinking in the home cage [55], whereas ERK1/2 activation has been suggested to play a role in keeping alcohol drinking in moderation [31,75]. These differences may reflect the differential role of ERK1/2 in different brain regions (i.e., amygdala in our present study, and mPFC or NAc in previous studies [31,54,76]), as well as the different processes targeted (memory reconsolidation in our study, and alcohol self-administration in the previous studies).

The essential role of ERK1/2 in the reconsolidation process has been demonstrated repeatedly in studies of fear memory reconsolidation [77,78,79,80,81]. Furthermore, previous studies have shown that systemic [46,47], intra-BLA [45], or intra-NAc [48] ERK1/2 inhibition in conjunction with drug-memory retrieval disrupts the reconsolidation of drug memories, reflected by suppressed cocaine and morphine-seeking behavior. Thus, our findings here expand these findings by directly showing that activation of ERK1/2 plays a critical role in alcohol memory reconsolidation. Of note, the Western blot analyses in the present study were conducted on whole amygdala tissues, without further segregation into the amygdala sub-regions. Moreover, ERK1/2 inhibition was conducted systemically. Thus, we cannot determine the precise amygdala subregion (e.g., BLA, CeA) that mediates these effects. Given our previous results, which localized mTORC1 activation to the CeA [19], it is plausible that this brain region also mediates the effects of ERK1/2 on alcohol memory reconsolidation; however, this assumption remains to be tested in future studies. Our present finding, that ERK1/2 is activated in the amygdala following alcohol memory retrieval, and that its inhibition disrupts alcohol memory reconsolidation, taken together with our previous finding that mTORC1 inhibition in the amygdala yields similar results [19], led us to test whether both effects shared a common pathway. Specifically, as ERK1/2 is a positive regulator of mTORC1 [29,30,42], ERK1/2 inhibition may have impaired mTORC1 activation in the amygdala, which is essential for the reconsolidation process [19]. Surprisingly, we found that the systemic administration of a MEK1/2 inhibitor had a trend towards increasing the phosphorylation of S6, used as a marker for mTORC1 activity [19,32,53]. Therefore, it is not likely that ERK1/2 and mTORC1 share the same mechanism of action in alcohol memory reconsolidation disruption.

Importantly, ERK1/2 is a key component of various intracellular signaling pathways that underlie synaptic and neuronal plasticity [82,83,84,85]. ERK1/2 signaling is crucial for the activation of several transcription factors that were implicated in memory reconsolidation, including ETS-like gene-1 (ELK-1) [47] and CREB [47,64,65,66]. Indeed, cocaine and morphine memory retrieval led to increased activation of ERK1/2 and CREB in the NAc, which was suppressed by ERK1/2 inhibition [47,48]. Moreover, we have recently demonstrated that retrieval of alcohol memories, by exposing mice to the alcohol-associated context, led to increased activation of CREB in the mPFC and dorsal hippocampus, and was followed by elevated mRNA expression of the CREB transcriptional targets, *Arc* and *Zif268* [64]. Moreover, the post-retrieval downregulation of *Arc* in the dorsal hippocampus disrupted the alcohol memory reconsolidation [64]. Indeed, we found that the increased ERK1/2 activation, which was observed in the amygdala two hours following the alcohol memory retrieval, was accompanied by increased activation of CREB. Therefore, it is plausible that the impaired reconsolidation caused by ERK1/2 inhibition was mediated via the MAPK-CREB pathway, rather than the mTORC1 pathway.

### 3.2. CRMP2 Inhibition Impairs Alcohol Memory Reconsolidation

Our findings suggest that CRMP2 plays a role in the reconsolidation of alcohol memories. Specifically, we found that post-retrieval inhibition of CRMP2 by the FDA-approved drug lacosamide impaired the reconsolidation of alcohol memories, as reflected by the suppression of relapse to alcohol drinking.

Systemic inhibition of CRMP2 using lacosamide was previously shown to decrease alcohol binge drinking [86] and to prevent the reinstatement of alcohol-conditioned place preference [53] when the treatment was administered shortly before the behavioral test. Here, we demonstrate that lacosamide also interferes with the reconsolidation of alcohol memories, consequently reducing relapse to alcohol drinking a day after the injection. CRMP2 serves as a multifunctional protein, mainly known for its ability to promote neurite outgrowth by regulating microtubule dynamics [50,52,87]. However, as the CRMP2-tubulin interaction was shown to be mediated via CRMP2 phosphorylation [49,88,89,90], and we found that alcohol memory retrieval did not affect CRMP2 phosphorylation, it is very unlikely that CRMP2-mediated alteration in tubulin polymerization is involved in the reconsolidation of alcohol memories.

A note of caution is due here, as lacosamide is an anti-epileptic drug shown to reduce neuronal excitability by enhancing slow inactivation of voltage-gated sodium channels [62,91]. Therefore, we cannot exclude the possibility that the observed effect of lacosamide on the reconsolidation of alcohol memories may also be mediated by other mechanisms targeted by this drug.

It has previously been demonstrated that the reinstatement of alcohol place preference by an alcohol prime injection increased CRMP2 levels in the NAc [53]. Similarly, an increased expression of CRMP2 was also observed in the mPFC following exposure to cocaine-associated cues during the reinstatement test for operant cocaine-seeking [92]. Contrary to these previous reports, we did not observe any change in CRMP2 protein level or in its phosphorylation following the mere exposure to an alcohol cue (i.e., memory retrieval). These conflicting findings raise the possibility that the exposure to drug-related cues alone, in the absence of the drug itself or the behavioral expression of drug seeking, may not be sufficient to induce changes in CRMP2 expression.

We also found that GSK3β inhibition did not affect the reconsolidation of alcohol memories. Interestingly, GSK3β inhibition was previously reported to disrupt the reconsolidation of fear- [93] and drug- [35,36,39,94] memories. Our findings therefore suggest that unlike other drugs of abuse, alcohol memory reconsolidation appears not to depend on GSK3β signaling, at least in the current experimental setting.

To conclude, our findings underscore the crucial role of ERK1/2 and CRMP2 in the reconsolidation of alcohol-associated memories and subsequent relapse to alcohol drinking. Conversely, signaling pathways involving AKT and GSK3β do not appear to play a role in this context for alcohol memory retrieval and reconsolidation. Importantly, the capacity of lacosamide, an FDA-approved CRMP2 inhibitor, to disrupt the reconsolidation of alcohol memories holds significant translational implications. Importantly lacosamide, SB 216763, and SL-327 were all administered systemically in the present study. Thus, the effects of these manipulations cannot be attributed to a specific brain region. To affiliate the causal role of the investigated signaling pathways in the process of alcohol memory reconsolidation to a specific brain region, a localized administration of the treatments into specific brain regions is required.

## 4. Materials and Methods

### 4.1. Animals

Male and female Wistar rats (160–240 gr at the beginning of experiments 1–4, and 220–390 gr at the beginning of experiment 5) were bred at the Tel Aviv University animal facility. Animals were individually housed under a 12-h light-dark cycle (lights on at 7 a.m.) with food and water available ad libitum. Animals were weighed once a week to control weight loss. All experimental protocols were approved by and conformed to the guidelines of the Institutional Animal Care and Use Committee of Tel Aviv University, and the NIH guidelines (animal welfare assurance number A5010-01). All efforts were made to minimize the number of animals used.

### 4.2. Drugs and Reagents

The alcohol-drinking solution was prepared by diluting ethanol absolute (0005250502000) (BioLab, Jerusalem, Israel) to a 20% (*v*/*v*) solution with tap water. Isoflurane was obtained from Piramal Critical Care (Bethlehem, PA, USA). Rabbit antibodies for pAKT-Thr308 (9275), pAKT-Ser473 (4046), total AKT (9272), pGSK3β-Ser9 (9323), total pGSK3β (9315), pCRMP2-Thr514 (9397), total CRMP2 (9393), pERK1/2-Thr202/Tyr204 (4370), total ERK1/2 (9102), pCREB-Ser133 (9198), total CREB (9197), pS6-Ser235/236 (2211), total S6 (2217), as well as anti-rabbit (7074) and anti-mouse (7076) horseradish peroxidase (HRP)-linked antibodies, were obtained from Cell Signaling Technology (Denvers, MA, USA). A mouse antibody for glyceraldehyde 3-phosphate dehydrogenase (GAPDH) (sc-32233) was obtained from Santa Cruz Biotechnology (Santa Cruz, CA, USA). A protease/phosphatase inhibitor cocktail (5872) was purchased from Cell Signaling Technology (Danvers, MA, USA). Pierce BCA protein assay kit (23227) and SuperSignal chemiluminescent substrate (34578) were purchased from Thermo Fisher Scientific (Waltham, MA, USA). Immobilon-P PVDF membrane (IPVH00005) was purchased from Merck (Rehovot, Israel). BLUeye prestained protein ladder (PM007-0500) was purchased from BIO-HELIX (New Taipei City, Taiwan). 12% Criterion TGX precast midi protein gels (5671044) and 4× Laemmli sample buffer (1610747) were purchased from Bio-Rad Laboratories (Rishon Le Zion, Israel). The CRMP2 inhibitor, lacosamide (465325000) (Thermo Scientific Chemicals, Waltham, MA, USA), was solubilized in saline. The GSK3β inhibitor, SB 216763 (HY-12012) (MedChemExpress, Monmouth Junction, NJ, USA), was solubilized in DMSO. The MEK1/2 inhibitor, SL-327 (S1066) (Selleck Chemicals, Houston, TX, USA), was solubilized in DMSO.

### 4.3. Behavioral Procedure

Intermittent access to 20% alcohol in 2-bottle choice (IA2BC)

This procedure was conducted as we previously described [95,96,97,98,99,100,101,102,103,104,105]. Briefly, following a week of habituation to individual cages, rats were trained to consume 20% alcohol solution in the intermittent Access 2-bottle-choice procedure (IA2BC). Animals were given three 24-h sessions of free access to 2-bottle choice per week (tap water and 20% alcohol *v*/*v*) on Sundays, Tuesdays, and Thursdays. Alcohol drinking sessions were followed by a 24- or 48-h deprivation period, in which the animals received only water, producing repeated cycles of intoxication and withdrawal. The position (left or right) of the alcohol and water bottles was alternated between the sessions to control for side preference. Alcohol exposure in this protocol was shown to produce high levels of alcohol intake and blood alcohol concentration, especially in the first hours of drinking [95,96,99,106,107]. Water and alcohol bottles were weighed before and after each alcohol-drinking session, and consumption levels were normalized to body weight. In the IA2BC experiments that included injections (experiments 2–4), the rats were handled weekly and received several sessions of injection handling with saline the week before the injection of the pharmacological substance.

Memory retrieval after IA2BC: After 9–10 weeks of training, rats were subjected to 10 days of abstinence. On the 11th day of abstinence, alcohol memory retrieval was performed in the rats’ home cages with 10 min exposure to an empty bottle of alcohol with its tip covered with a drop of alcohol serving as an odor-taste cue, as was previously described [19]. Control rats were left untouched in their home cages.

### 4.4. Western Blot

Western blot procedure was conducted as previously described [64]. Briefly, immediately after dissection, brain tissues were homogenized in a radioimmunoprecipitation assay (RIPA) buffer containing: 150 mM NaCl, 5 mM EDTA, 50 mM Tris HCl, 1% Triton X-100, 0.5% sodium deoxycholate, 0.1% sodium dodecyl sulfate (SDS), and protease and phosphatase inhibitors. The homogenates were then centrifuged at 14,600 rpm and the supernatants were collected and stored at −80 °C until use. Prior to sample preparations, protein concentrations were determined using BCA assay. An equal amount of each sample (30 μg) was mixed with 4× Laemmli sample buffer that was added with dithiothreitol (DTT) (50 mM), and the mixture was boiled for five min at 100 °C. Samples were separated on a 12% polyacrylamide gel and were then transferred onto a PVDF membrane at 100 V for one hour. Membranes were blocked for one hour at room temperature with 5% bovine serum albumin (BSA) in TBST Tris-buffered saline and 0.1% Tween 20 (TBST), and were then incubated overnight at 4 °C with the appropriate primary antibody (pAKT-Thr308 1:500, pAKT-Ser473 1:2000, pGSK3β 1:1000, pCRMP2 1:800, pERK1/2 1:500, pCREB 1:700, pS6 1:1000, GAPDH 1:10,000). Membranes were washed with TBST and probed with the appropriate HRP-conjugated secondary antibodies (anti-rabbit 1:3000, anti-mouse 1:5000) for one hour at room temperature. After washing with TBST, the bound antibodies were visualized using enhanced chemiluminescent (ECL) HRP substrate and captured with the VILBER Fusion FX imaging system. Membranes were then incubated for one hour in a stripping buffer containing 62.5 mM Tris, 100 mM β-mercaptoethanol, and 2% SDS, which was heated to 50 °C. The membranes were then washed extensively with double distilled water (DDW) and with TBST, blocked for one hour at room temperature, and were re-incubated overnight at 4 °C with the appropriate primary antibody (total AKT 1:1000, total GSK3β 1:1000, total CRMP2 1:1000, total ERK1/2 1:1000, total CREB 1:700, total S6 1:1000). Membranes were again washed, incubated with the anti-rabbit secondary antibody, and visualized. Band intensities were quantified using the ImageJ software (NIH), version 1.53k (see Supplementary File for images of the full-length bands). The optical density values of the phosphorylated proteins’ immunoreactivity were normalized to those of the total proteins, and immunoreactivity values of total CRMP2 were normalized to those of GAPDH. The phosphorylated/total protein expression of the experimental groups was calculated as a percentage of the control group.

### 4.5. Experimental Design and Statistical Analysis

The allocation of rats to the experimental groups was conducted based on their average alcohol drinking to create groups with approximately equal alcohol drinking levels. Sex was distributed approximately equally across the experiments and was initially analyzed as a factor. The analyses in all the experiments did not yield an interaction between sex and other factors. Therefore, data were collapsed across this factor.

#### 4.5.1. Experiment 1—Effects of Alcohol Memory Retrieval on the Activation of the PI3K-AKT and ERK1/2 Signaling Pathways

Rats were trained to voluntarily consume alcohol in the IA2BC protocol, as described above. After nine weeks of drinking, the rats were subjected to 10 days of abstinence. On the 11th day of the abstinence period, the rats’ alcohol memory was retrieved using an odor-taste cue, as described above. Control rats did not undergo the memory retrieval procedure and were left untouched. mPFC, NAc, whole amygdala, and OFC tissues were collected 30 and 60 min after the alcohol memory retrieval procedure and were processed for a western-blot analysis. The samples were examined for changes in the phosphorylation levels of AKT, GSK3β, CRMP2, and ERK1/2, as well as changes in the total protein levels of CRMP2. The expression level for each target in each brain region was normalized to the no-retrieval group. The expression data of each target were analyzed by one-way ANOVA with a between factor of Group (no-retrieval, retrieval—30 min, retrieval—60 min).

#### 4.5.2. Experiment 2—Effects of CRMP2 Inhibition on Alcohol Memory Reconsolidation

Rats were trained in the IA2BC protocol for nine weeks, followed by an abstinence period and a procedure of alcohol memory retrieval, as described in Experiment 1. Immediately after the memory retrieval, the rats were injected with the CRMP2 inhibitor, lacosamide (20 mg/kg, i.p., injection volume 1 mL/kg), or with a vehicle solution. The dose was based on Liu et al. [86]. The rats’ drinking behavior was tested 24 h later. After the drinking test, the rats were subjected to two additional weeks of drinking training, followed by 10 days of abstinence. On the 11th day of abstinence, the rats were treated again with lacosamide or with vehicle, but without a prior procedure of alcohol memory retrieval. The treatment was done in a counterbalanced manner, i.e., rats that received lacosamide in the post-retrieval test, received vehicle in the no-retrieval test and vice versa. Three rats that displayed drinking levels lower than 2 gr/kg (according to the average of the last three sessions prior to the abstinence) did not proceed to the no-retrieval test. In each test the following parameters were collected: alcohol consumption (gr/kg), alcohol preference (volume of alcohol consumed/volume of alcohol + water consumed), and water consumption (mL/kg). The data of each parameter in each test were analyzed by an unpaired *t*-test (vehicle vs. lacosamide).

#### 4.5.3. Experiment 3—Effects of GSK3β Inhibition on Alcohol Memory Reconsolidation

Rats were trained in the IA2BC protocol for 10 weeks, followed by an abstinence period and a procedure of alcohol memory retrieval, as described in Experiment 1. Immediately after the memory retrieval, the rats were injected with the GSK3β inhibitor, SB 216763 (20 mg/kg, i.p., injection volume 0.5 mL/kg), or with a vehicle solution. SB 216763 dose was based on Shi et al. [35]. The rats’ drinking behavior was tested 24 h later. The parameters that were collected for the analysis were the same as in Experiment 2. The data of each parameter were analyzed by an unpaired *t*-test (vehicle vs. SB 216763).

#### 4.5.4. Experiment 4—Effects of ERK1/2 Inhibition on Alcohol Memory Reconsolidation

Rats were trained in the IA2BC protocol for 10 weeks, followed by an abstinence period and a procedure of alcohol memory retrieval, as described in Experiment 1. Immediately after the memory retrieval, the rats were injected with the inhibitor of ERK1/2 upstream regulator MEK1/2, SL-327 (50 mg/kg, i.p., injection volume 0.6 mL/kg), or with a vehicle solution. SL-327 dose was based on Sarantis et al. [67]. The rats’ drinking behavior was tested 24 h later and 14 days later. Between the two tests, the rats were abstinent from alcohol. After the second drinking test, the rats were subjected to three additional weeks of drinking training, followed by 10 days of abstinence, and an additional no-retrieval drinking test, as was done in Experiment 2. The parameters that were collected for the analyses were the same as in Experiment 2. The data of each parameter in each test was analyzed by an unpaired *t*-test (vehicle vs. SL-327). Rats were then subjected again to three additional weeks of drinking, followed by an abstinence period. On the 11th day of the abstinence period, the rats’ alcohol memory was retrieved, and whole amygdala tissues were collected two hours later and were processed for western blot analysis. Control rats did not undergo the memory retrieval procedure and were left untouched. The samples were examined for changes in the phosphorylation levels of ERK1/2 and CREB. The phosphorylation levels of the retrieval group were normalized to the no-retrieval group. Data were analyzed by an unpaired *t*-test (no-retrieval vs. retrieval).

#### 4.5.5. Experiment 5—Effects of Systemic Administration of the MEK1/2 Inhibitor on the Phosphorylation of ERK1/2 and S6 in the Amygdala

Naïve rats were separated into individual cages and after one week, during which they were handled for injections, the rats were injected with SL-327 (50 mg/kg. i.p., injection volume 0.6 mL/kg). Control rats were injected with a vehicle solution. Tissues of the whole amygdala were collected 90 min afterward [68,69] and were processed for western blot analysis. The samples were examined for changes in the phosphorylation levels of ERK1/2 and S6. The phosphorylation levels of the SL-327 group were normalized to the vehicle group. Data were analyzed by an unpaired *t*-test (vehicle vs. SL-327).

## Data Availability

The data presented in this study are available on request from the corresponding author. The Western blot raw data are contained within the article and Supplementary Material.

## Supplementary Materials

The following supporting information can be downloaded at: https://www.mdpi.com/article/10.3390/ijms25105478/s1.
