# Supplementary material for: Inhibition of ERK1/2 or CRMP2 Disrupts Alcohol Memory Reconsolidation and Prevents Relapse in Rats
Source: Int J Mol Sci. 2024 May 17;25(10):5478. doi: 10.3390/ijms25105478 (PMC11122309; doi:10.3390/ijms25105478)

Supplementary Figure S1

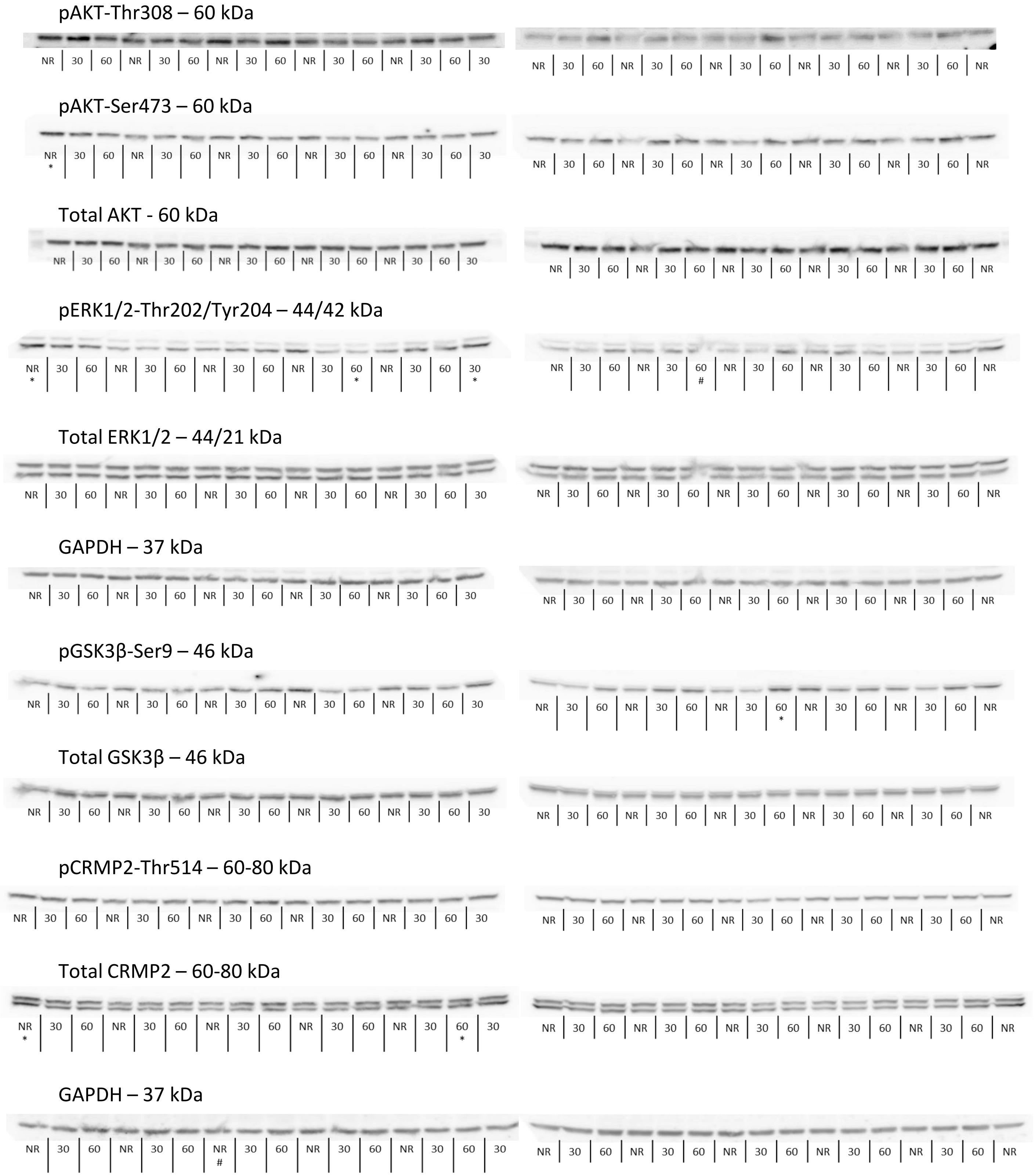

Figure S1. Western-blot visualization of phospho-protein and total-protein levels in the mPFC (Experiment 1). The tissues were collected 30 min (30) or 60 min (60) after alcohol memory retrieval, or without a prior retrieval procedure (NR)**.** Phospho-protein levels of AKT (Thr308, Ser473), GSK3β (Ser9), CRMP2 (Thr514) and ERK1/2 (Thr202/Tyr204) were normalized to the total protein immunoreactivity. Total protein levels of CRMP2 were normalized to GAPDH. AKT and ERK1/2 antibodies were blotted on the same set of membranes, and therefore share the GAPDH levels. GSK3β and CRMP2 were blotted on a different set of membranes. * Samples that were identified as outliers to their experimental group (z>2) and were therefore removed from the analysis. # Distorted bands that were removed from the analysis.

Supplementary Figure S2

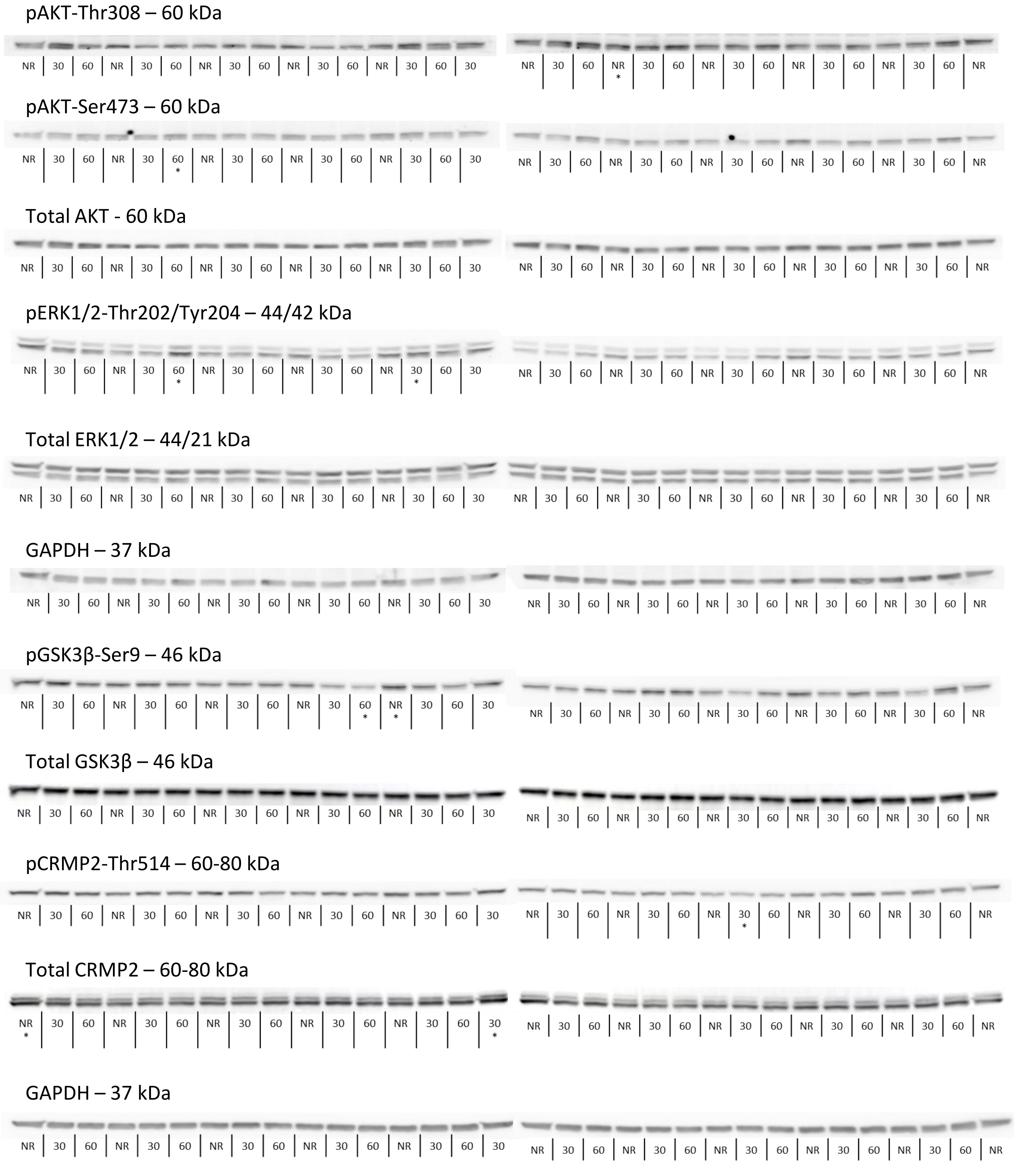

Figure S2. Western-blot visualization of phospho-protein and total-protein levels in the NAc (Experiment 1). The tissues were collected 30 min (30) or 60 min (60) after alcohol memory retrieval, or without a prior retrieval procedure (NR). Phospho-protein levels of AKT (Thr308, Ser473), GSK3β (Ser9), CRMP2 (Thr514) and ERK1/2 (Thr202/Tyr204) were normalized to the total protein immunoreactivity. Total protein levels of CRMP2 were normalized to GAPDH. AKT and ERK1/2 antibodies were blotted on the same set of membranes, and therefore share the GAPDH levels. GSK3β and CRMP2 were blotted on a different set of membranes. * Samples that were identified as outliers to their experimental group (z>2) and were therefore removed from the analysis.

Supplementary Figure S3

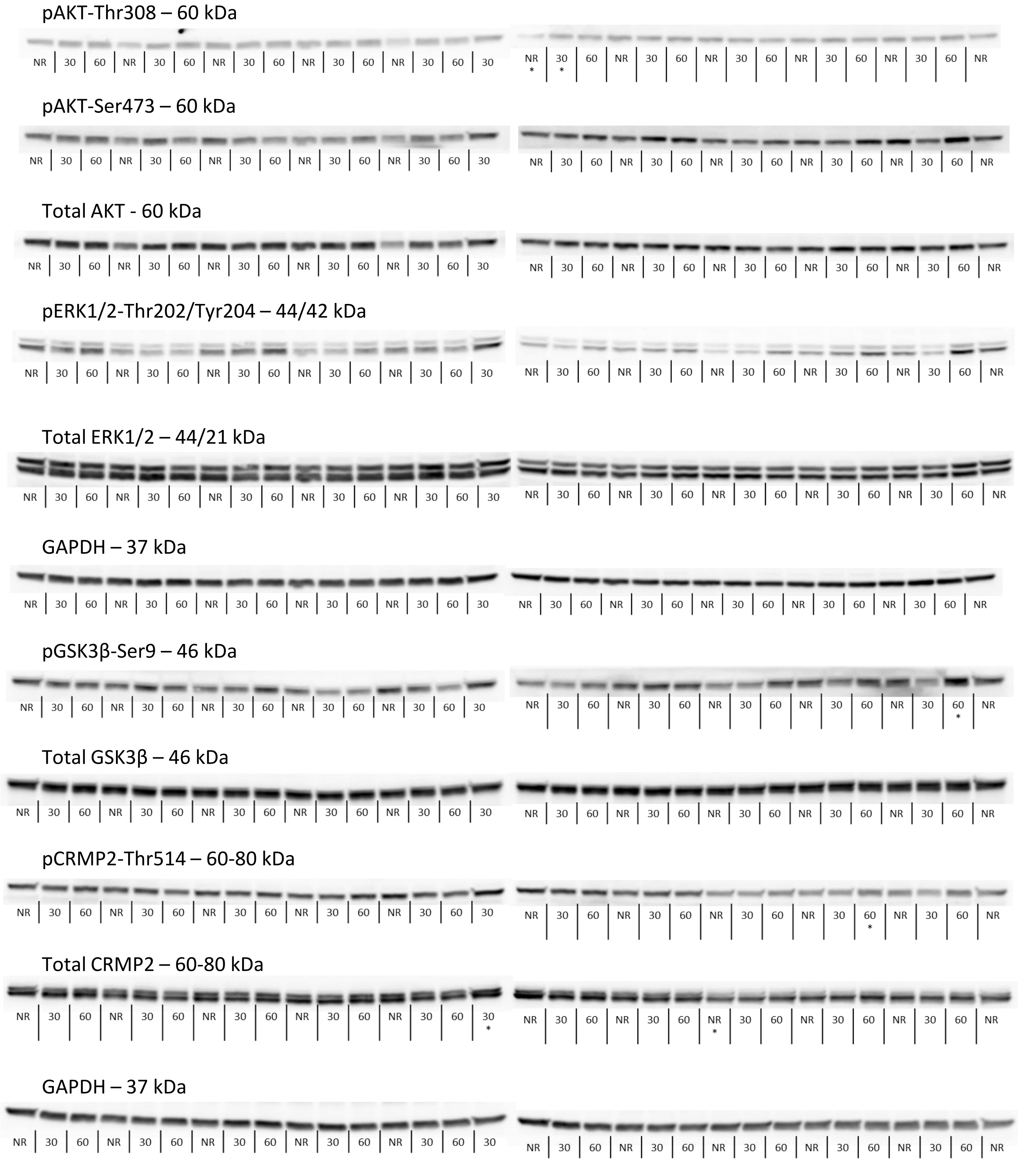

Figure S3. Western-blot visualization of phospho-protein and total-protein levels in the amygdala (Experiment 1). The tissues were collected 30 min (30) or 60 min (60) after alcohol memory retrieval, or without a prior retrieval procedure (NR)**.** Phospho-protein levels of AKT (Thr308, Ser473), GSK3β (Ser9), CRMP2 (Thr514) and ERK1/2 (Thr202/Tyr204) were normalized to the total protein immunoreactivity. Total protein levels of CRMP2 were normalized to GAPDH. AKT and ERK1/2 antibodies were blotted on the same set of membranes, and therefore share the GAPDH levels. GSK3β and CRMP2 were blotted on a different set of membranes. * Samples that were identified as outliers to their experimental group (z>2) and were therefore removed from the analysis.

Supplementary Figure S4

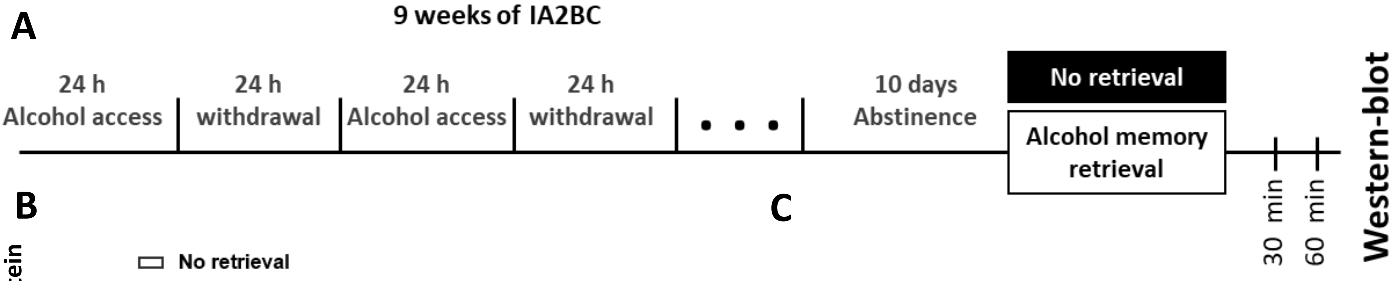

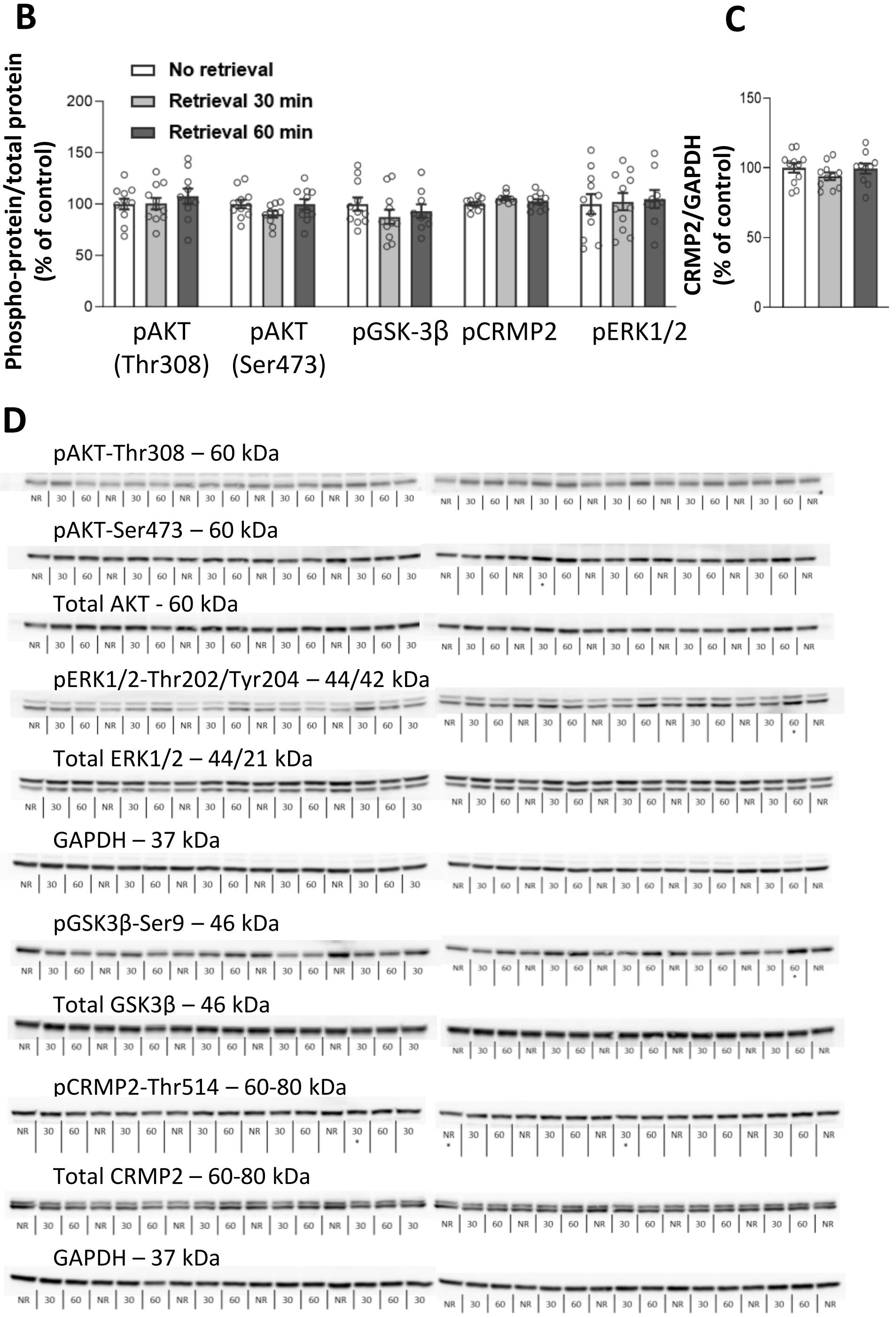

Figure S4. Alcohol memory retrieval does not affect the PI3K-AKT signaling pathway in the OFC. A. Experimental design and timeline. Rats consumed alcohol in the intermittent access to 20% alcohol in a 2-bottle choice paradigm for 9 weeks, followed by 10 days of abstinence. After the abstinence period, alcohol memory was retrieved using an odor-taste cue. Tissues were collected 30 or 60 min after the retrieval. Phospho- and total-protein levels were determined by western blot. **B.** Phospho-protein levels of AKT (Thr308, Ser473), glycogen synthase kinase 3β (GSK3β) (Ser9), collapsin response mediator protein-2 (CRMP2) (Thr514) and extracellular regulated kinase1/2 (ERK1/2) (Thr202, Tyr204), normalized to the total protein immunoreactivity. (one-way ANOVA: pAKT (Thr308) [$F(2,29)=0.48$, $p=0.623$), pAKT (Ser473) [$F(2,28)=1.76$, $p=0.19$], pGSK3β [$F(2,28)=0.941$, $p=0.402$], pCRMP2 [$F(2,26)=1.97$, $p=0.159$], pERK1/2 [$F(2,28)=0.07$, $p=0.929$]. **C.** CRMP2 levels normalized to glyceraldehyde 3-phosphate dehydrogenase (GAPDH) (one-way ANOVA: $F(2,29)=1.06$, $p=0.359$). Bar graphs are mean±SEM expressed as percentage of No retrieval controls. n=9-11 (4-5 males, 4-6 females) per group. **D.** Western-blot visualization of phospho-protein and total-protein levels. AKT and ERK1/2 antibodies were blotted on the same set of membranes, and therefore share the GAPDH levels. GSK3β and CRMP2 were blotted on a different set of membranes. * Samples that were identified as outliers to their experimental group ($z>2$) and were therefore removed from the analysis.

Supplementary Figure S5

A

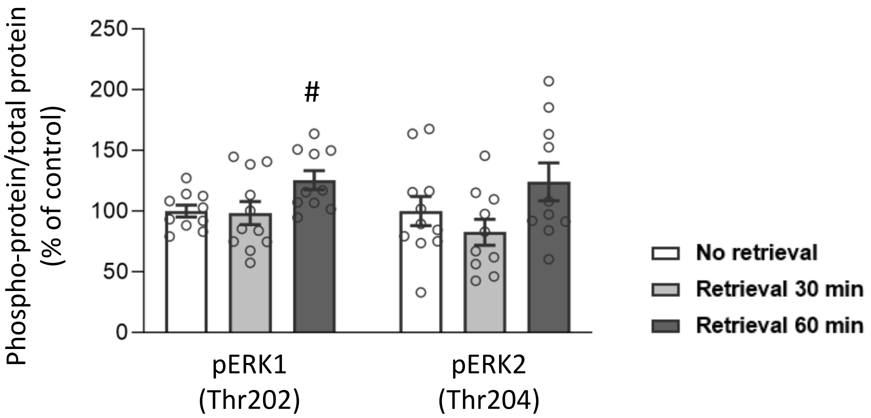

B

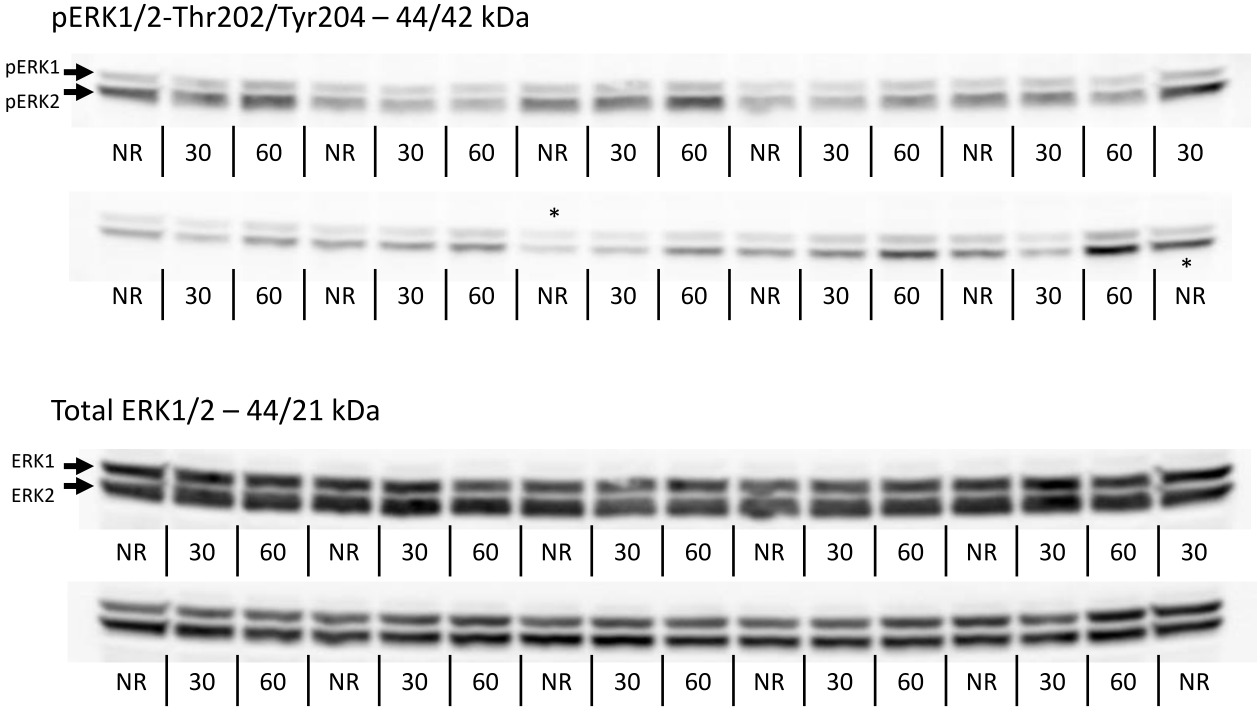

Figure S5. Alcohol memory retrieval increases ERK1 phosphorylation in the amygdala (Experiment 1). A. Separate analyses of ERK1 and ERK2 Phosphorylation levels in amygdala samples collected 30 and 60 min after alcohol memory retrieval. Phospho-protein levels of extracellular regulated kinase1/2 (ERK1/2) (Thr202, Tyr204) were normalized to the total protein immunoreactivity. Increased phosphorylation of ERK1 was observed 60 min after the retrieval (one-way ANOVA: pERK1: $F(2,28)=3.75$, $p=0.036$; Dunnett post hoc: No-retrieval vs. retrieval 30-min, $p=0.982$, No-retrieval vs. retrieval 60-min, $p=0.057$; pERK2: $F(2,28)=2.56$, $p= 0.096$). Bar graphs represent mean±SEM of the percent of change from the control No retrieval group. n=10-11 (4-5 males, 5-6 females). # p = 0.057 **B.** Western-blot visualization of phospho-protein and total-protein levels. * Samples that were identified as outliers to their experimental group (z>2) and were therefore removed from the analysis.

Supplementary Figure S6

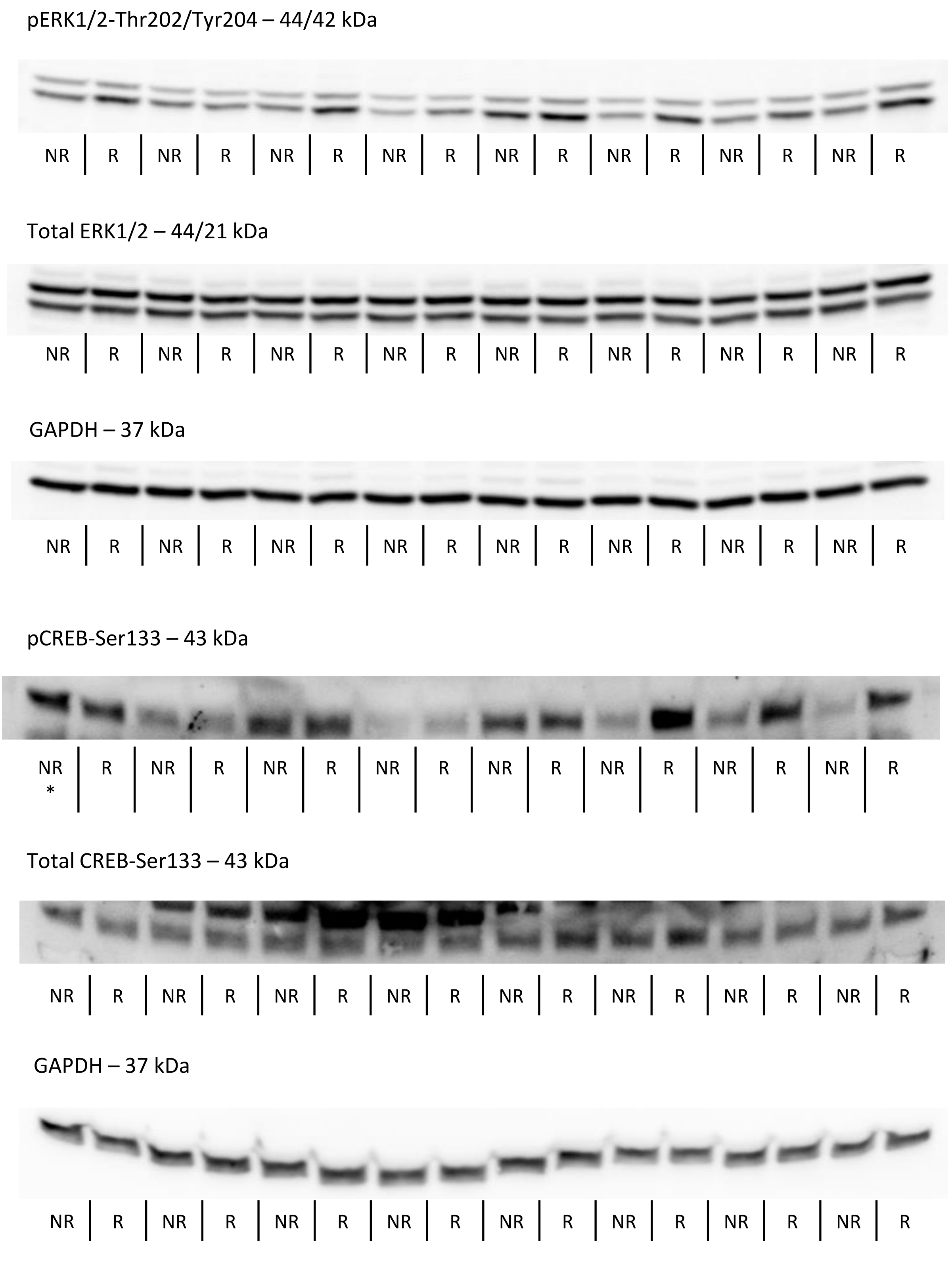

Figure S6. Western-blot visualization of phospho-protein and total-protein levels in the amygdala (Experiment 4). The tissues were collected two hours after alcohol memory retrieval (R), or without a prior retrieval procedure (NR)**.** Phospho-protein levels of ERK1/2 (Thr202/Tyr204) and CREB (Ser133) were normalized to the total protein immunoreactivity. ERK1/2 antibodies were blotted on one membrane and CREB antibodies were blotted on a different set of membrane. * Sample that was identified as an outlier to its experimental group (z>2) and was therefore removed from the analysis

Supplementary Figure S7

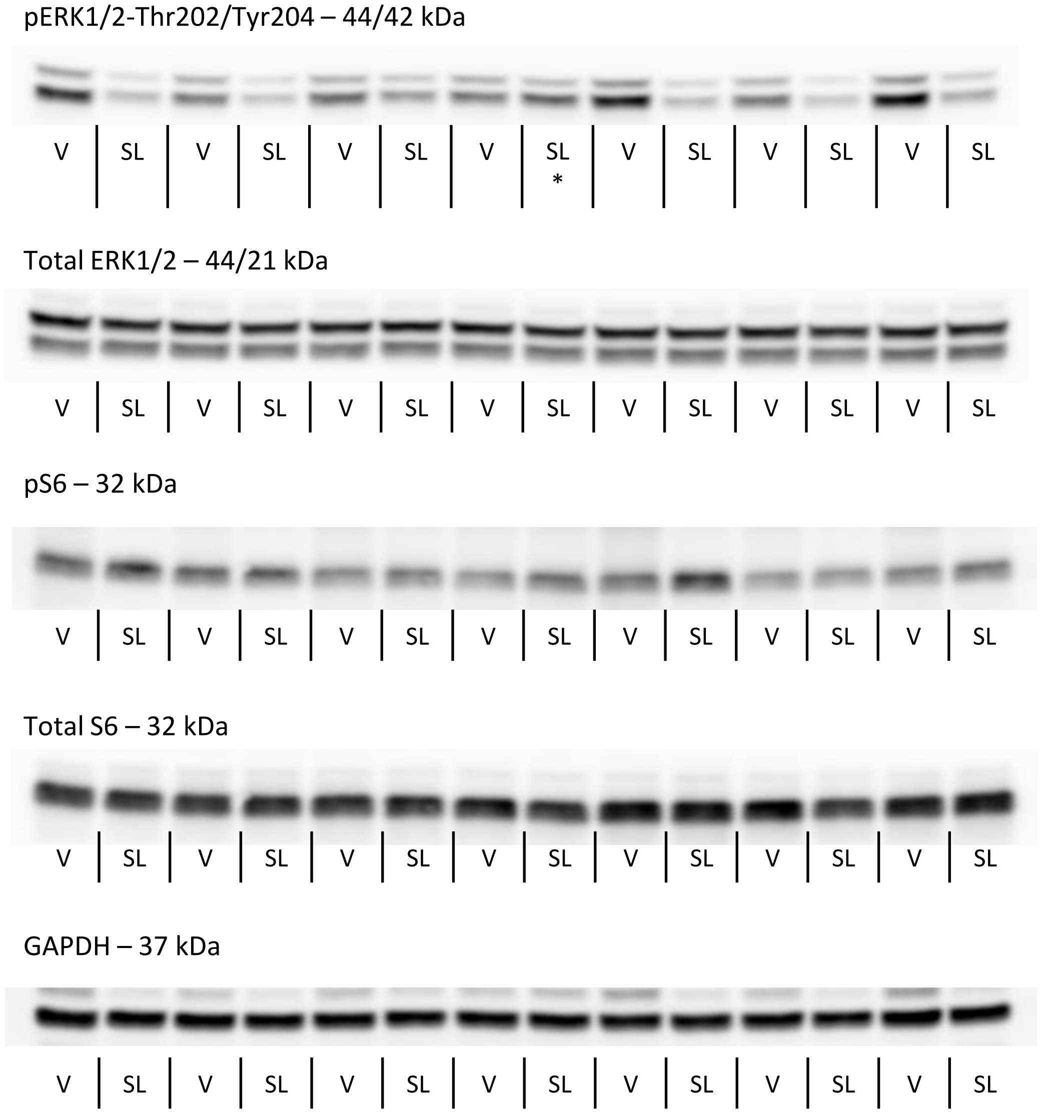

Figure S7. Western blot visualization of phospho-protein and total-protein levels in the amygdala (Experiment 5). The tissues were collected 90 min after injection of vehicle (V) or the MERK1/2 inhibitor SL-327 (50 mg/kg. i.p.) (SL). Phospho-protein levels of ERK1/2 (Thr202/Tyr204) and S6 (Ser235/236) were normalized to the total protein immunoreactivity. * Sample that was identified as an outlier to its experimental group (z>2) and was therefore removed from the analysis.

Experiment 1

Tissues were collected 30 min (30) or 60 min (60) after alcohol memory retrieval, or without a prior retrieval procedure (NR).

Due to the large number of samples, the samples were distributed across two gels / membranes.

AKT and ERK1/2 antibodies were blotted on the same set of membranes, and therefore share the GAPDH levels. GSK3β and CRMP2 were blotted on a different set of membranes.

The membranes were cut to segments prior to the blotting process, as illustrated below, therefore images of the complete membranes are not available.

* Samples that were identified as outliers to their experimental group (z>2), and were therefore removed from the analysis.

# Distorted bands that were removed from the analysis.

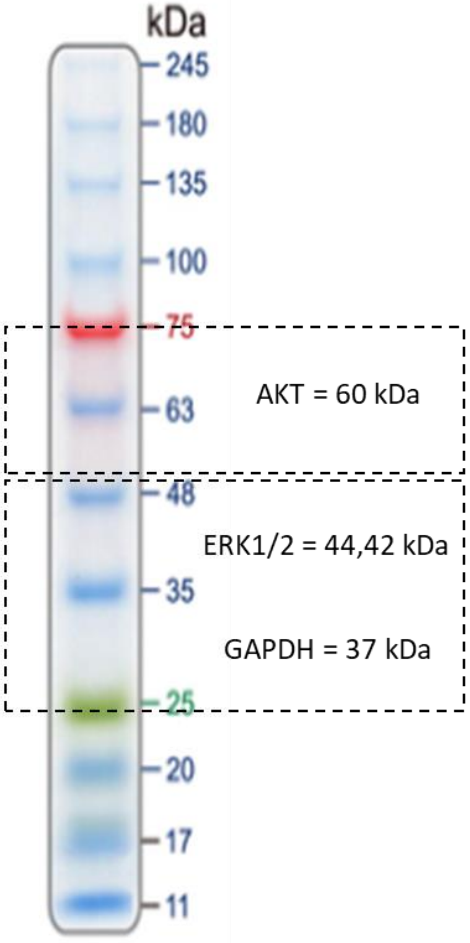

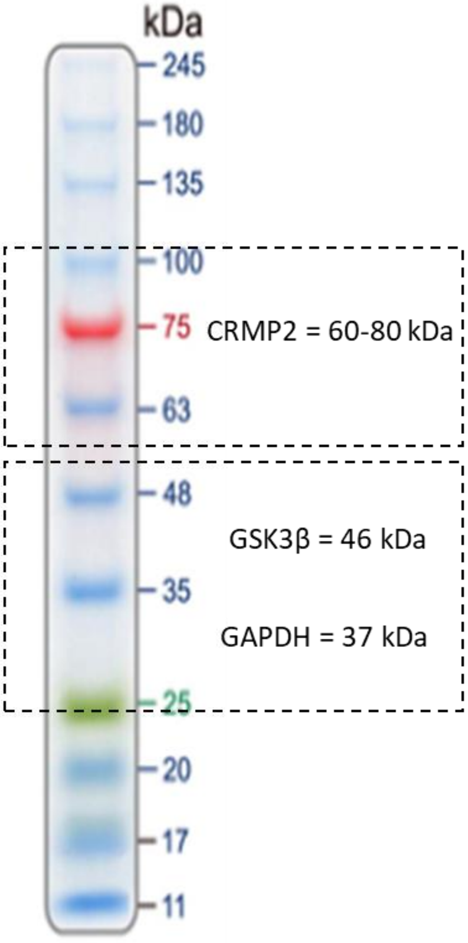

mPFC

pAKT-Thr308 – 60 kDa (Figures 1B and S1)

Lane #	1	2	3	4	5	6	7	8	9	10	11	12	13	14	15	16

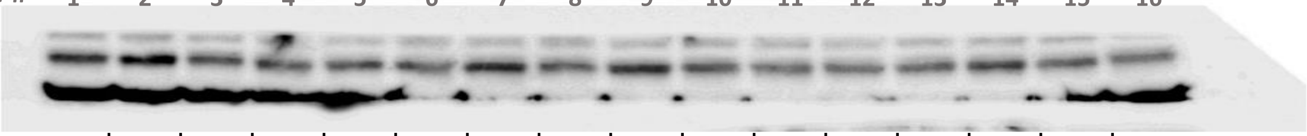

NR	30	60	NR	30	60	NR	30	60	NR	30	60	NR	30	60	30

17	18	19	20	21	22	23	24	25	26	27	28	29	30	31	32

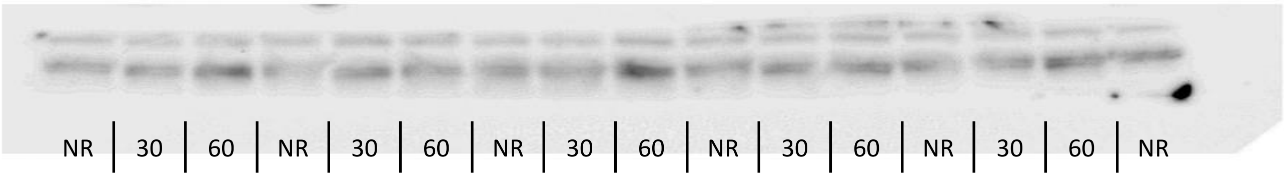

NR	30	60	NR	30	60	NR	30	60	NR	30	60	NR	30	60	NR

pAKT-Ser473 – 60 kDa (Figures 1B and S1)

Lane #	1	2	3	4	5	6	7	8	9	10	11	12	13	14	15	16

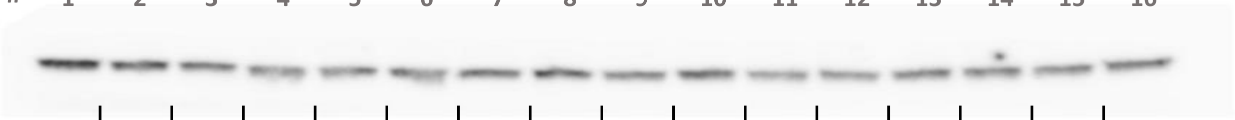

NR *	30	60	NR	30	60	NR	30	60	NR	30	60	NR	30	60	30

17	18	19	20	21	22	23	24	25	26	27	28	29	30	31	32

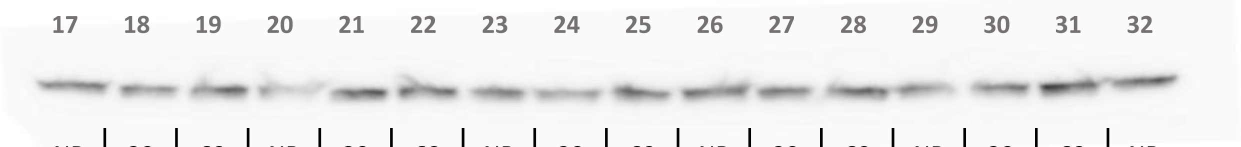

NR	30	60	NR	30	60	NR	30	60	NR	30	60	NR	30	60	NR

Total AKT - 60 kDa (Figures 1B and S1)

Lane #	1	2	3	4	5	6	7	8	9	10	11	12	13	14	15	16

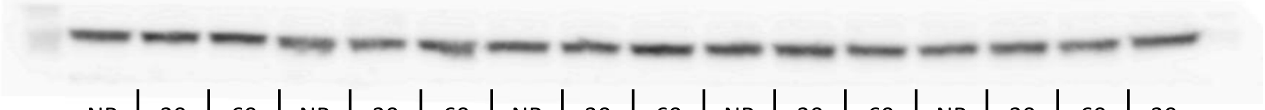

NR	30	60	NR	30	60	NR	30	60	NR	30	60	NR	30	60	30

17	18	19	20	21	22	23	24	25	26	27	28	29	30	31	32

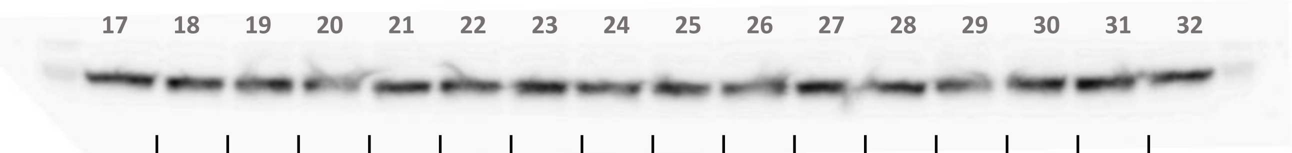

NR	30	60	NR	30	60	NR	30	60	NR	30	60	NR	30	60	NR

mPFC

pERK1/2-Thr202/Tyr204 – 44/42 kDa (Figures 4B and S1)

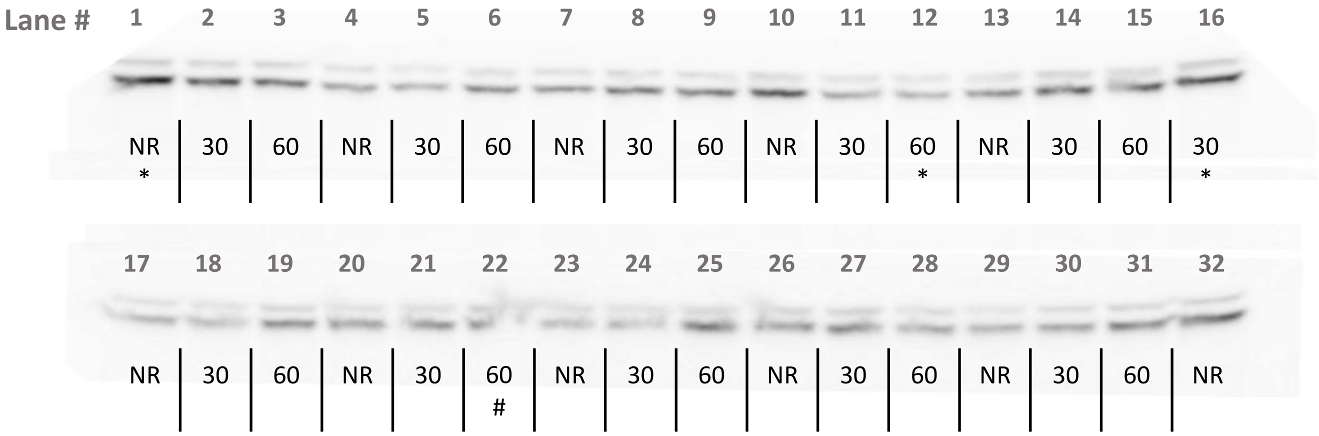

Total ERK1/2 – 44/21 kDa (Figures 4B and S1)

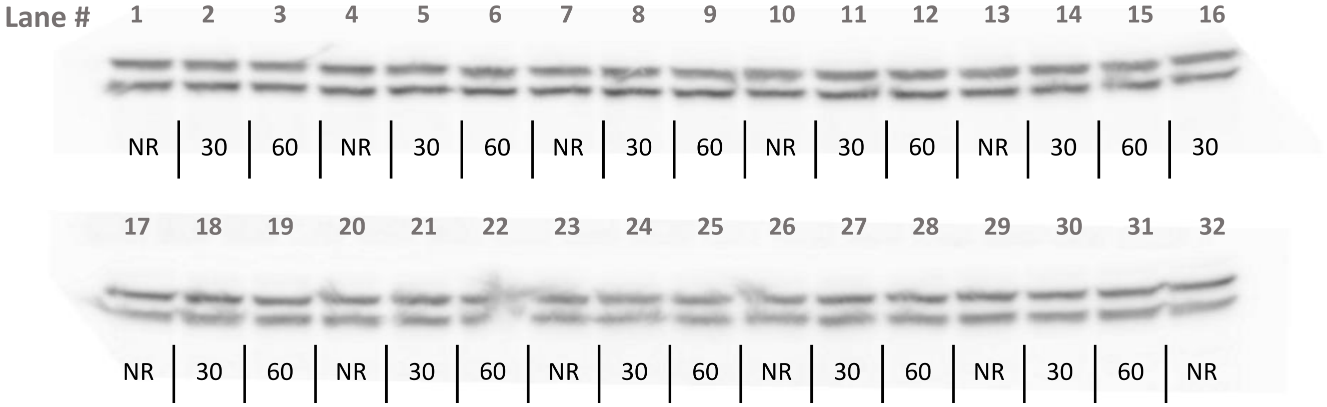

GAPDH – 37 kDa (Figure S1, not in the main figure)

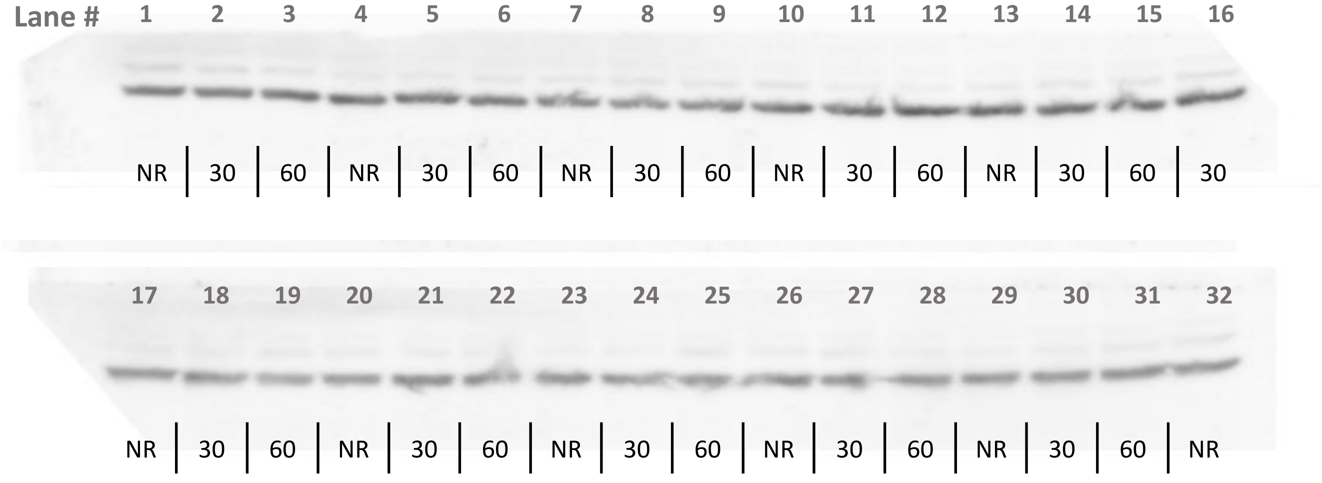

mPFC

pGSK3β-Ser9 – 46 kDa (Figures 1B and S1)

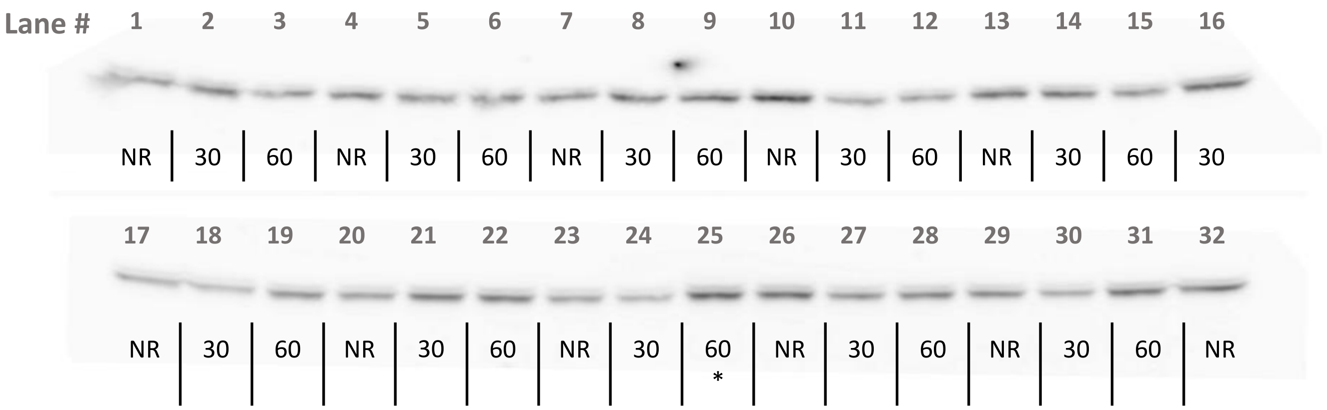

Total GSK3β – 46 kDa (Figures 1B and S1)

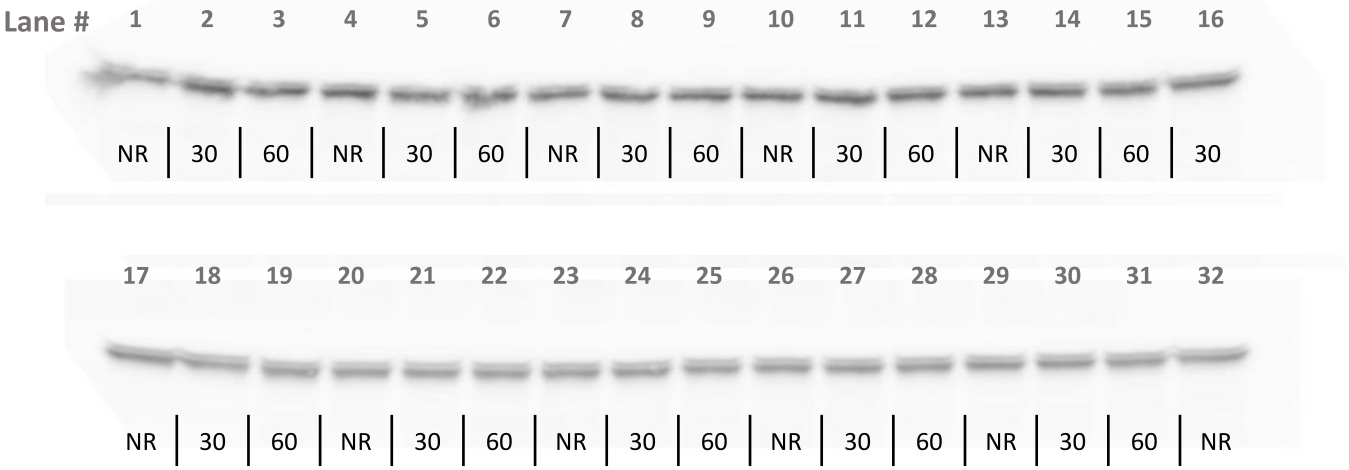

mPFC

pCRMP2-Thr514 – 60-80 kDa (Figures 1B and S1)

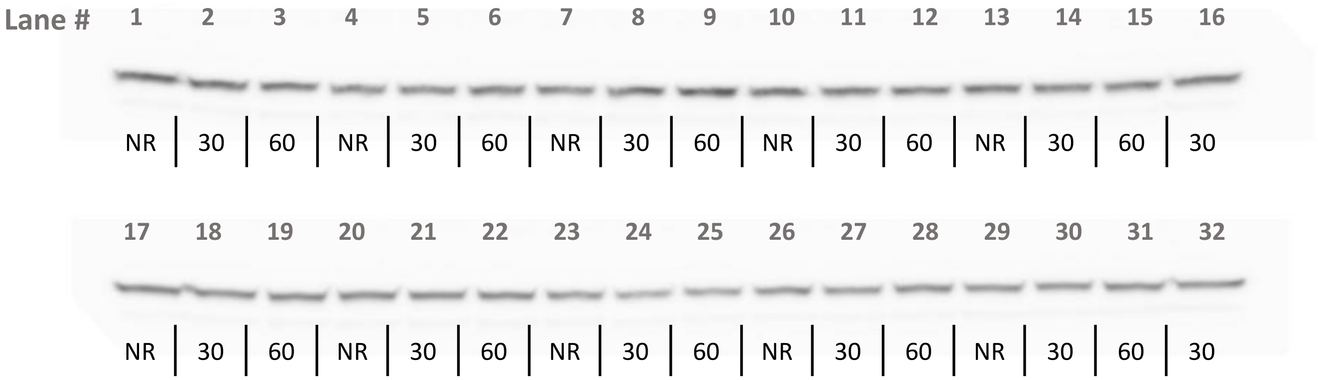

Total CRMP2 – 60-80 kDa (Figures 1C and S1)

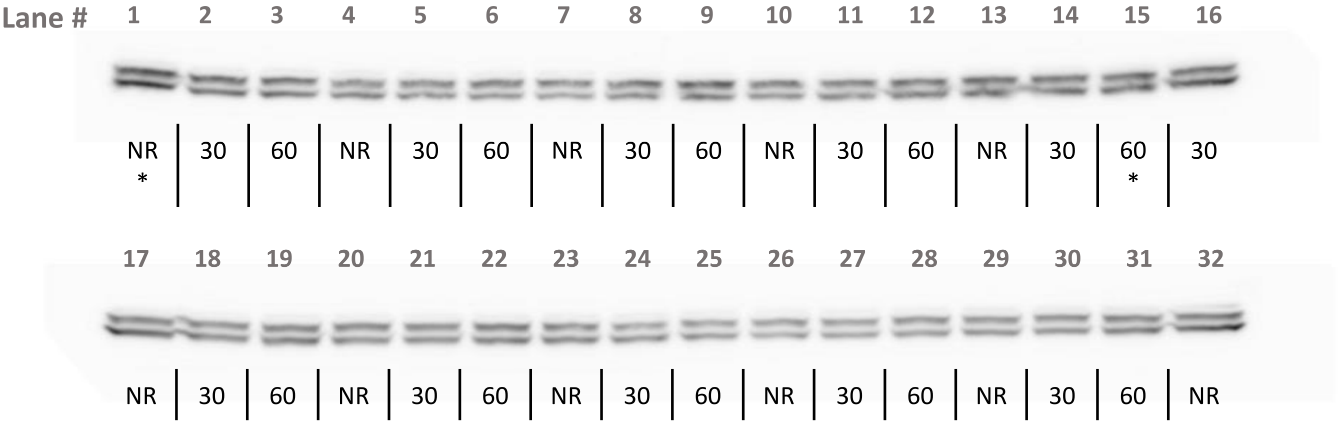

GAPDH – 37 kDa (Figures 1C and S1)

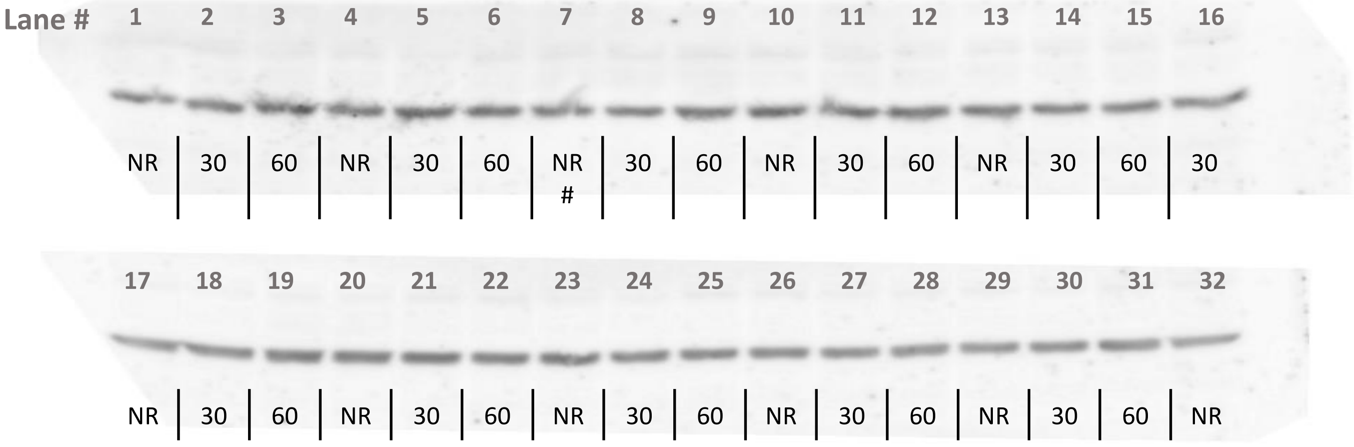

NAc

pAKT-Thr308 – 60 kDa (Figures 1D and S2)

Lane # 1 2 3 4 5 6 7 8 9 10 11 12 13 14 15 16

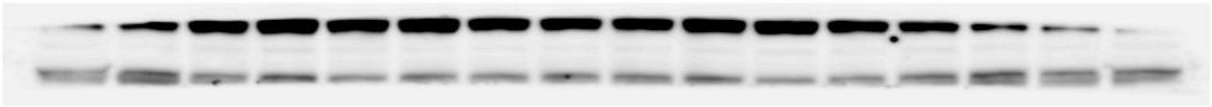

NR | 30 | 60 | NR | 30 | 60 | NR | 30 | 60 | NR | 30 | 60 | NR | 30 | 60 | 30

17 18 19 20 21 22 23 24 25 26 27 28 29 30 31 32

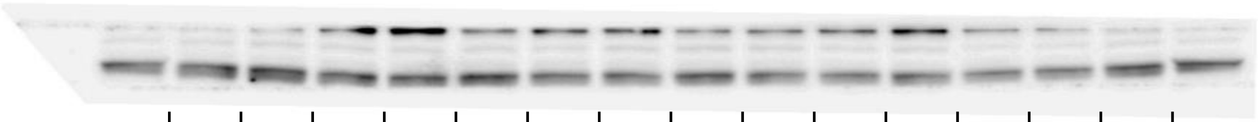

NR | 30 | 60 | NR * | 30 | 60 | NR | 30 | 60 | NR | 30 | 60 | NR | 30 | 60 | NR

pAKT-Ser473 – 60 kDa (Figures 1D and S2)

Lane # 1 2 3 4 5 6 7 8 9 10 11 12 13 14 15 16

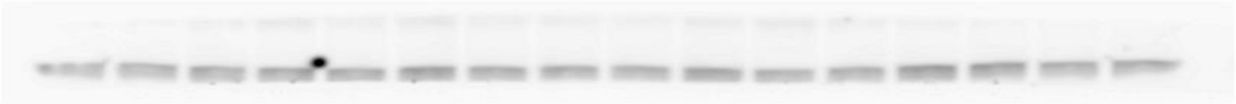

NR | 30 | 60 | NR | 30 | 60 * | NR | 30 | 60 | NR | 30 | 60 | NR | 30 | 60 | 30

17 18 19 20 21 22 23 24 25 26 27 28 29 30 31 32

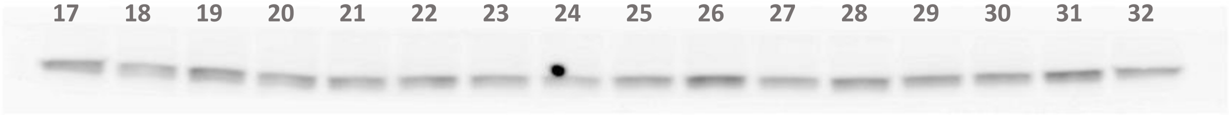

NR | 30 | 60 | NR | 30 | 60 | NR | 30 | 60 | NR | 30 | 60 | NR | 30 | 60 | NR

Total AKT - 60 kDa (Figures 1D and S2)

Lane # 1 2 3 4 5 6 7 8 9 10 11 12 13 14 15 16

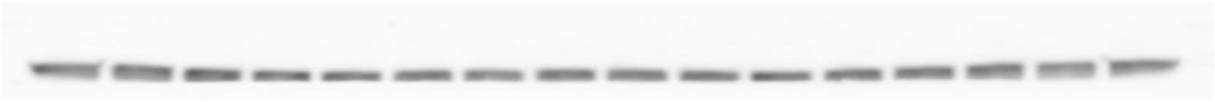

NR | 30 | 60 | NR | 30 | 60 | NR | 30 | 60 | NR | 30 | 60 | NR | 30 | 60 | 30

17 18 19 20 21 22 23 24 25 26 27 28 29 30 31 32

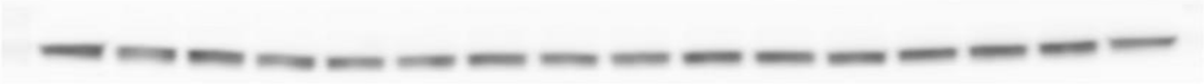

NR | 30 | 60 | NR | 30 | 60 | NR | 30 | 60 | NR | 30 | 60 | NR | 30 | 60 | NR

NAc

pERK1/2-Thr202/Tyr204 – 44/42 kDa (Figures 4B and S2)

Lane #	1	2	3	4	5	6	7	8	9	10	11	12	13	14	15	16
	NR	30	60	NR	30	60 *	NR	30	60	NR	30	60	NR	30 *	60	30

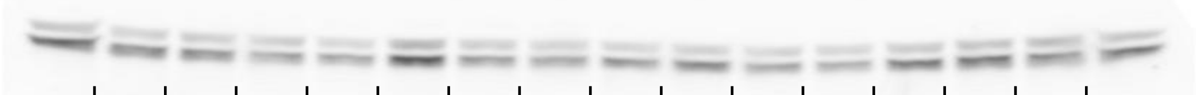

	17	18	19	20	21	22	23	24	25	26	27	28	29	30	31	32
	NR	30	60	NR	30	60	NR	30	60	NR	30	60	NR	30	60	NR

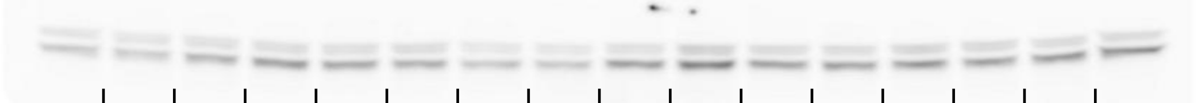

Total ERK1/2 – 44/21 kDa (Figures 4B and S2)

Lane #	1	2	3	4	5	6	7	8	9	10	11	12	13	14	15	16
	NR	30	60	NR	30	60	NR	30	60	NR	30	60	NR	30	60	30

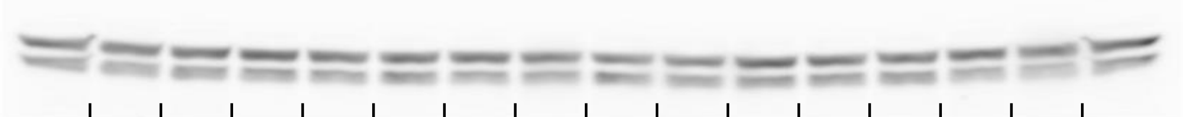

	17	18	19	20	21	22	23	24	25	26	27	28	29	30	31	32
	NR	30	60	NR	30	60	NR	30	60	NR	30	60	NR	30	60	NR

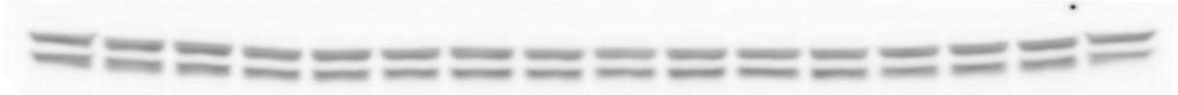

GAPDH – 37 kDa (Figure S2, not in the main figure)

Lane #	1	2	3	4	5	6	7	8	9	10	11	12	13	14	15	16
	NR	30	60	NR	30	60	NR	30	60	NR	30	60	NR	30	60	NR

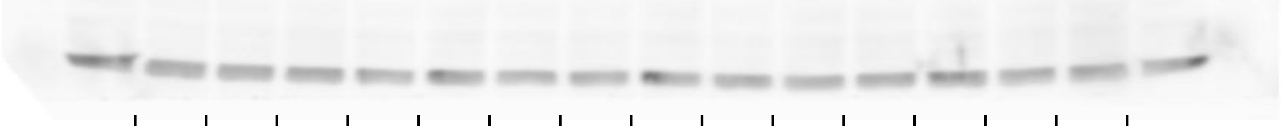

	17	18	19	20	21	22	23	24	25	26	27	28	29	30	31	32
	NR	30	60	NR	30	60	NR	30	60	NR	30	60	NR	30	60	NR

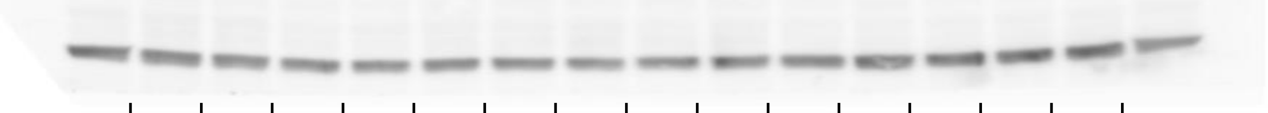

NAc

pGSK3β-Ser9 – 46 kDa (Figures 1D and S2)

Lane #	1	2	3	4	5	6	7	8	9	10	11	12	13	14	15	16

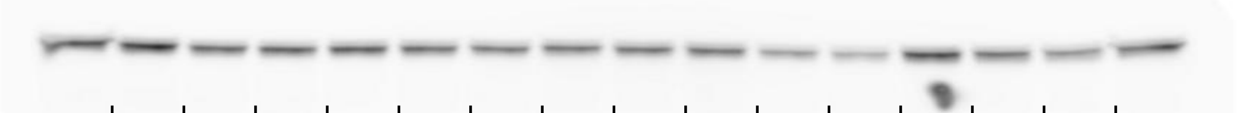

NR	30	60	NR	30	60	NR	30	60	NR	30	60 *	NR *	30	60	30

17	18	19	20	21	22	23	24	25	26	27	28	29	30	31	32

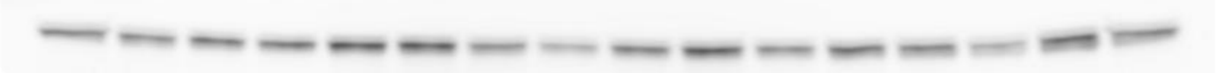

NR	30	60	NR	30	60	NR	30	60	NR	30	60	NR	30	60	NR

Total GSK3β – 46 kDa (Figures 1D and S2)

Lane #	1	2	3	4	5	6	7	8	9	10	11	12	13	14	15	16

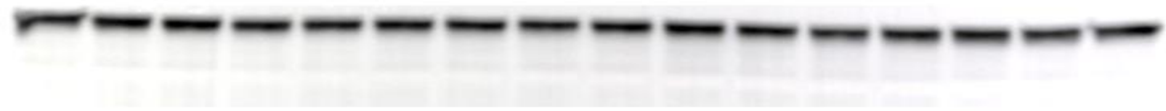

NR	30	60	NR	30	60	NR	30	60	NR	30	60	NR	30	60	30

17	18	19	20	21	22	23	24	25	26	27	28	29	30	31	32

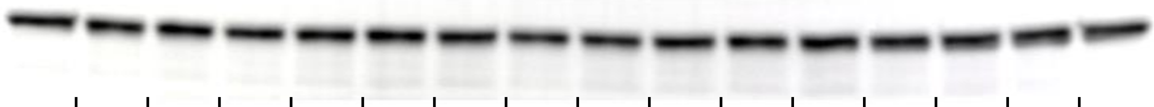

NR	30	60	NR	30	60	NR	30	60	NR	30	60	NR	30	60	NR

NAc

pCRMP2-Thr514 – 60-80 kDa (Figures 1D and S2)

Lane #	1	2	3	4	5	6	7	8	9	10	11	12	13	14	15	16
	NR	30	60	NR	30	60	NR	30	60	NR	30	60	NR	30	60	30

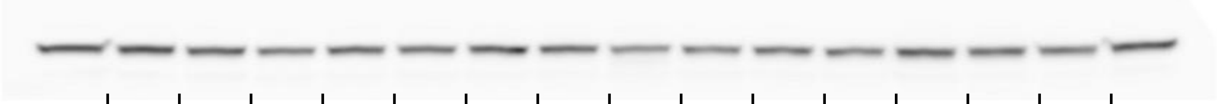

	17	18	19	20	21	22	23	24	25	26	27	28	29	30	31	32
	NR	30	60	NR	30	60	NR	30 *	60	NR	30	60	NR	30	60	NR

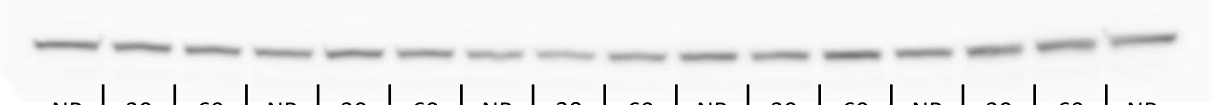

Total CRMP2 – 60-80 kDa (Figures 1E and S2)

Lane #	1	2	3	4	5	6	7	8	9	10	11	12	13	14	15	16
	NR *	30	60	NR	30	60	NR	30	60	NR	30	60	NR	30	60	30 *

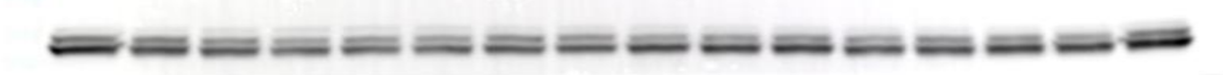

	17	18	19	20	21	22	23	24	25	26	27	28	29	30	31	32
	NR	30	60	NR	30	60	NR	30	60	NR	30	60	NR	30	60	NR

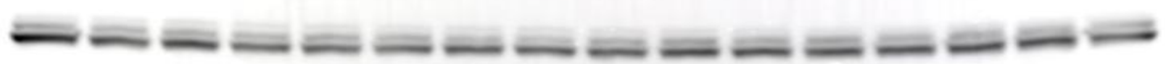

GAPDH – 37 kDa (Figures 1E and S2)

Lane #	1	2	3	4	5	6	7	8	9	10	11	12	13	14	15	16
	NR	30	60	NR	30	60	NR	30	60	NR	30	60	NR	30	60	30

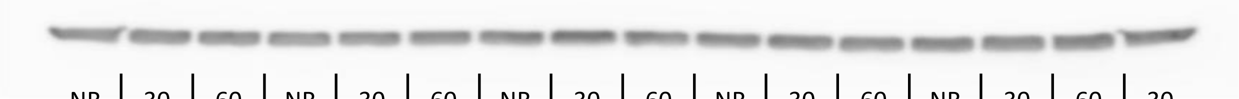

	17	18	19	20	21	22	23	24	25	26	27	28	29	30	31	32
	NR	30	60	NR	30	60	NR	30	60	NR	30	60	NR	30	60	NR

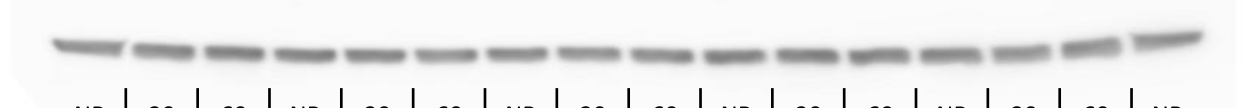

Amygdala

pAKT-Thr308 – 60 kDa (Figures 1F and S3)

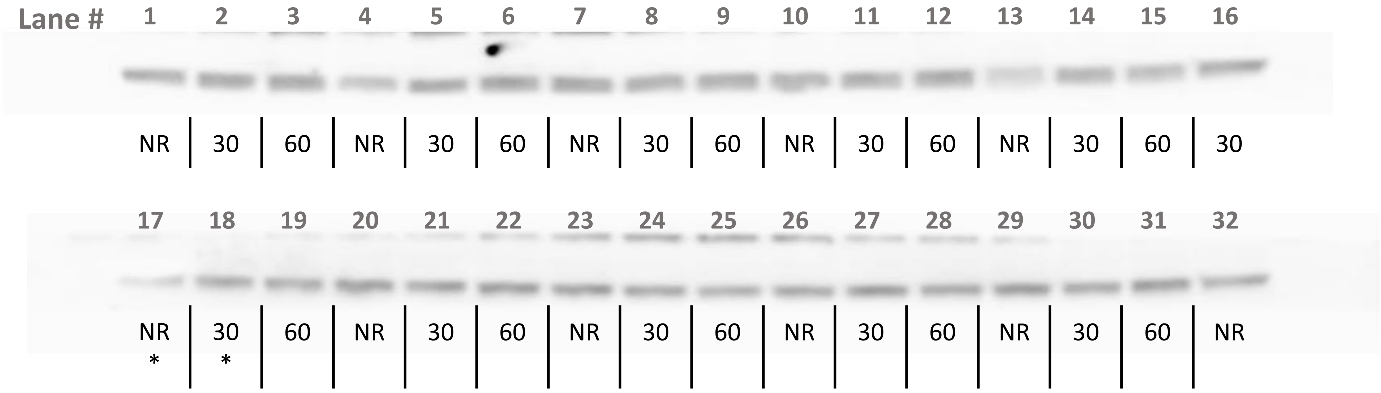

pAKT-Ser473 – 60 kDa (Figures 1F and S3)

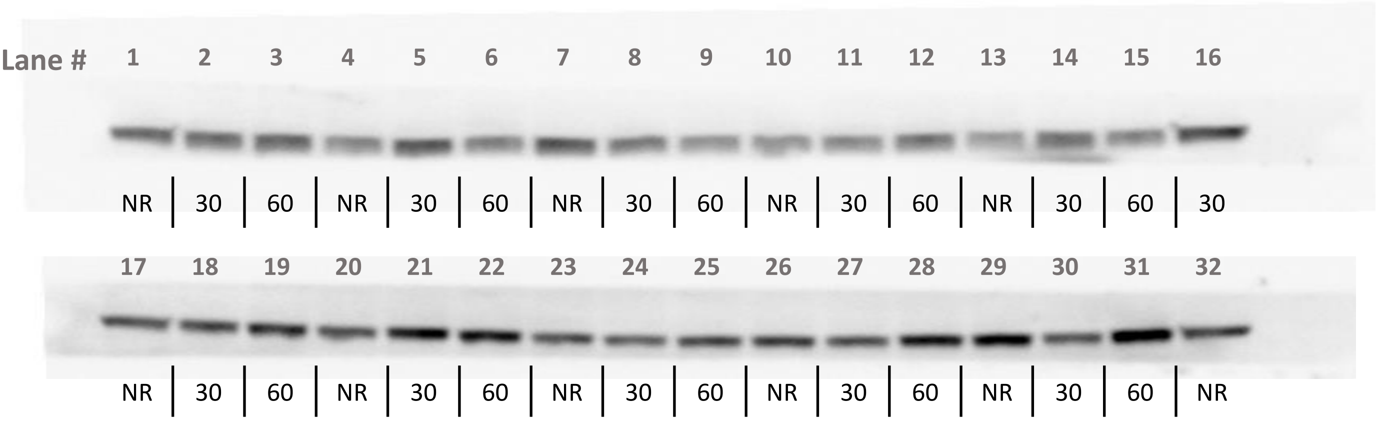

Total AKT - 60 kDa (Figures 1F and S3)

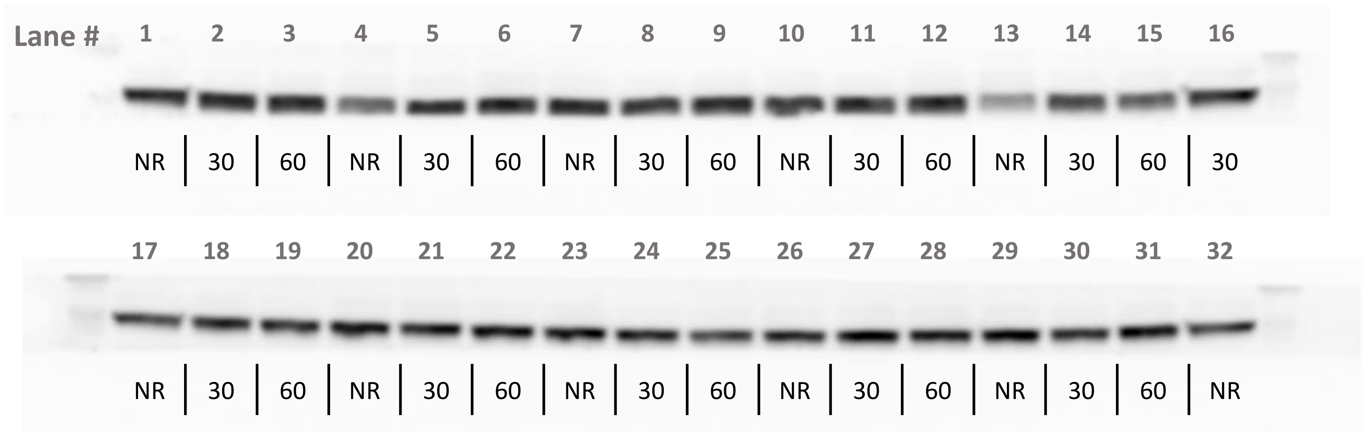

Amygdala

pERK1/2-Thr202/Tyr204 – 44/42 kDa (Figures 4B, S3 and S5)

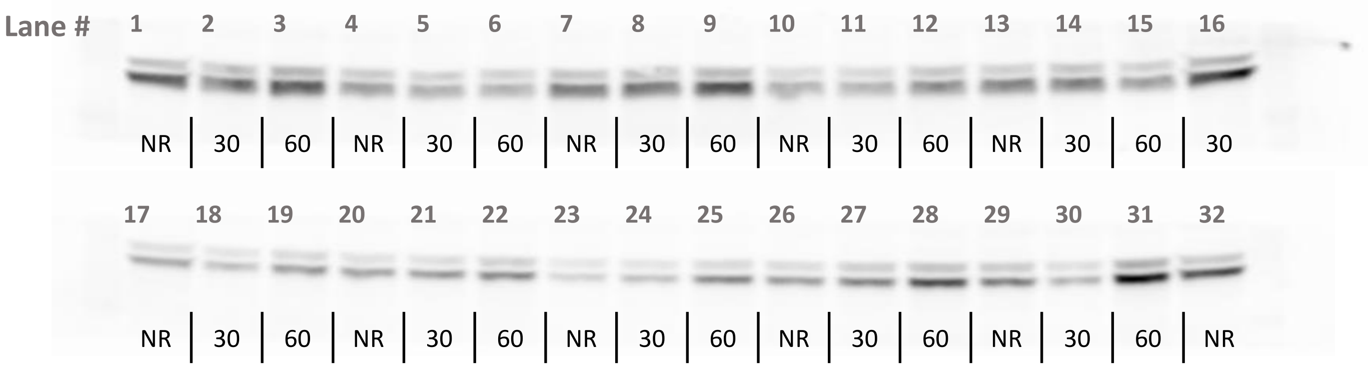

Total ERK1/2 – 44/21 kDa (Figures 4B, S3 and S5)

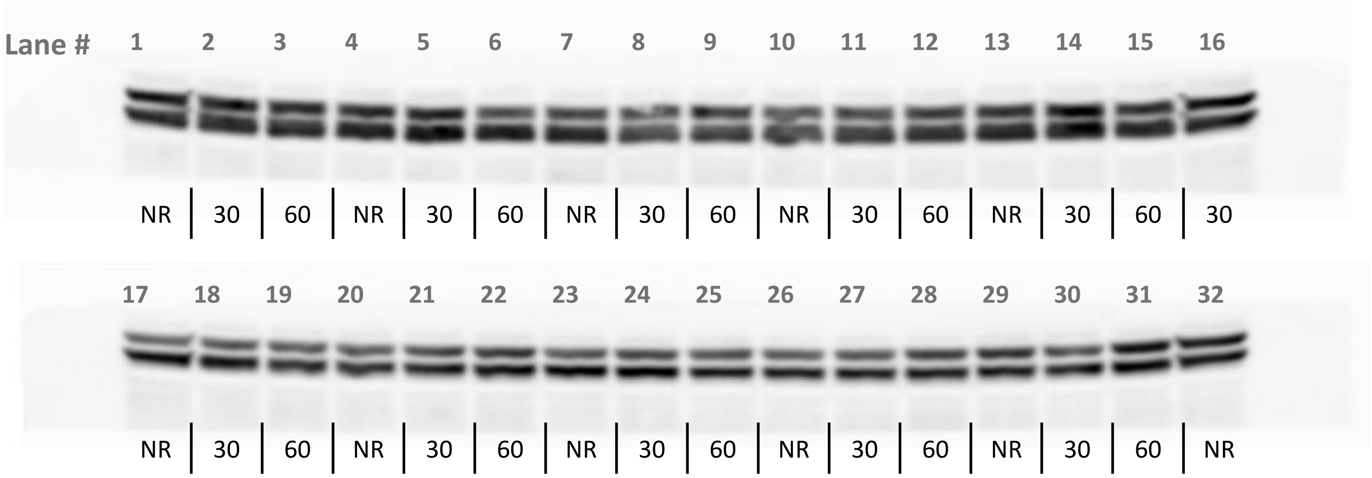

GAPDH – 37 kDa (Figure S3, not in the main figure)

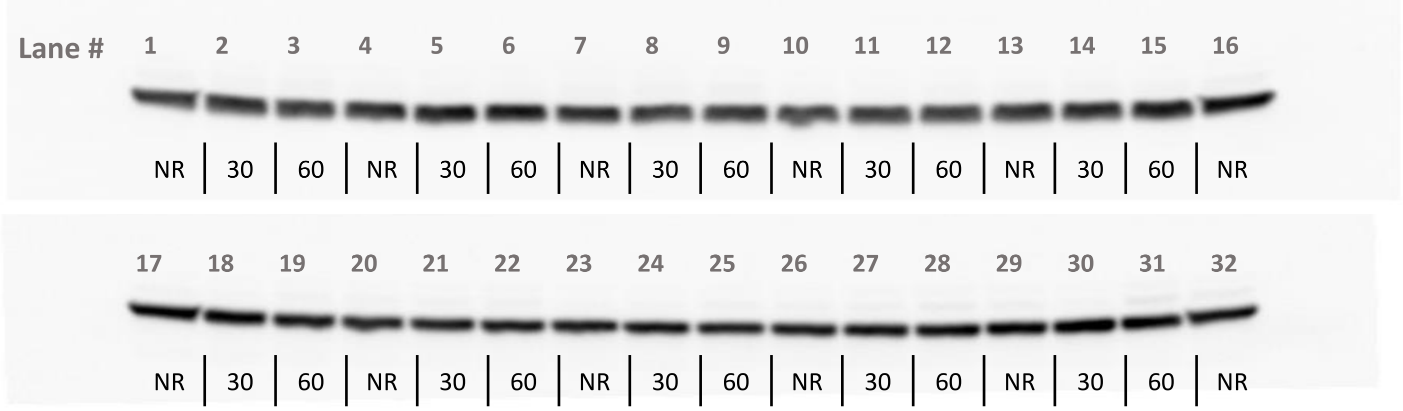

Amygdala

pGSK3β-Ser9 – 46 kDa (Figures 1F and S3)

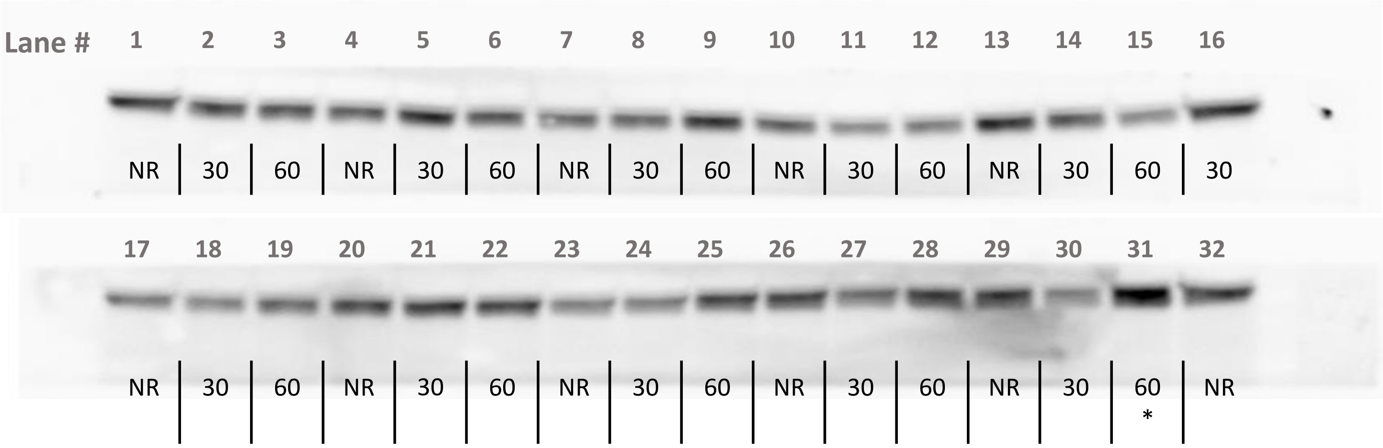

Total GSK3β – 46 kDa (Figures 1F and S3)

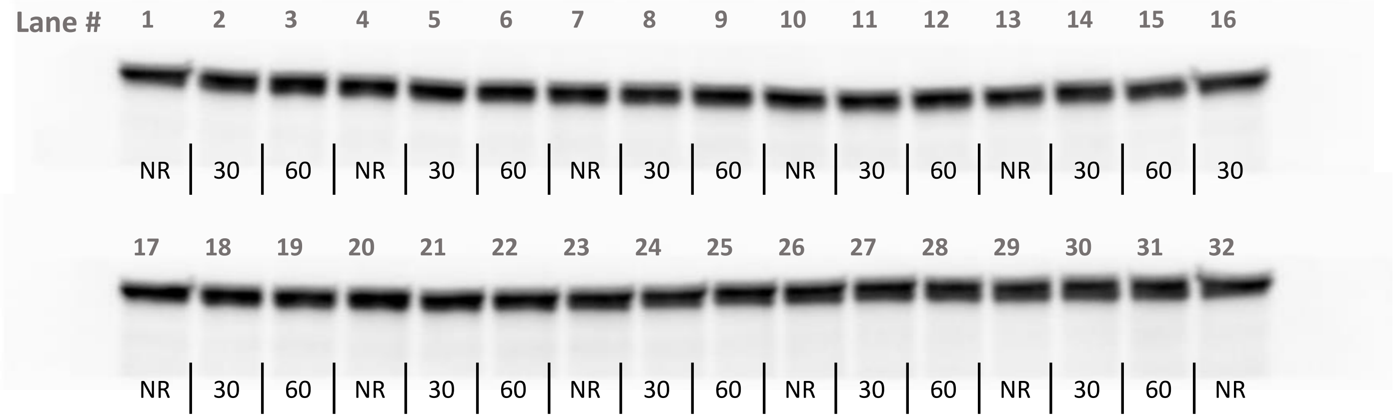

Amygdala

pCRMP2-Thr514 – 60-80 kDa (Figures 1F and S3)

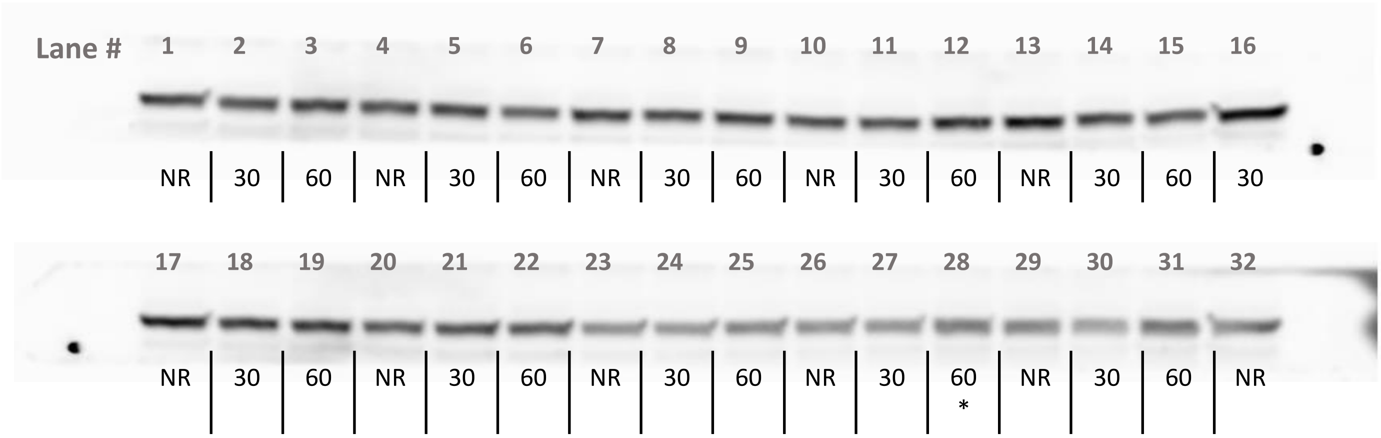

Total CRMP2 – 60-80 kDa (Figures 1G and S3)

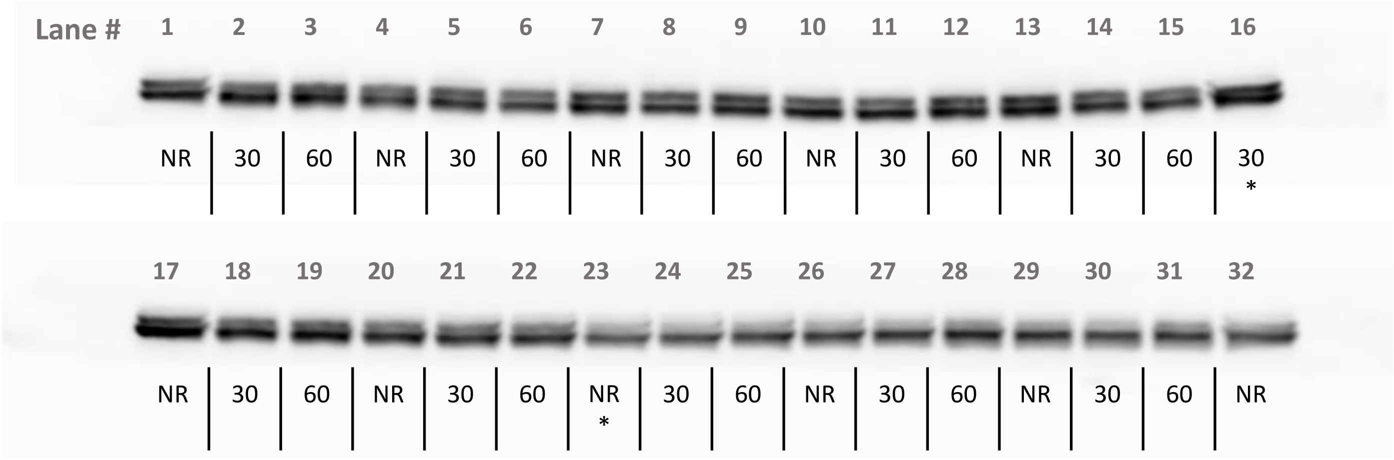

GAPDH – 37 kDa (Figures 1G and S3)

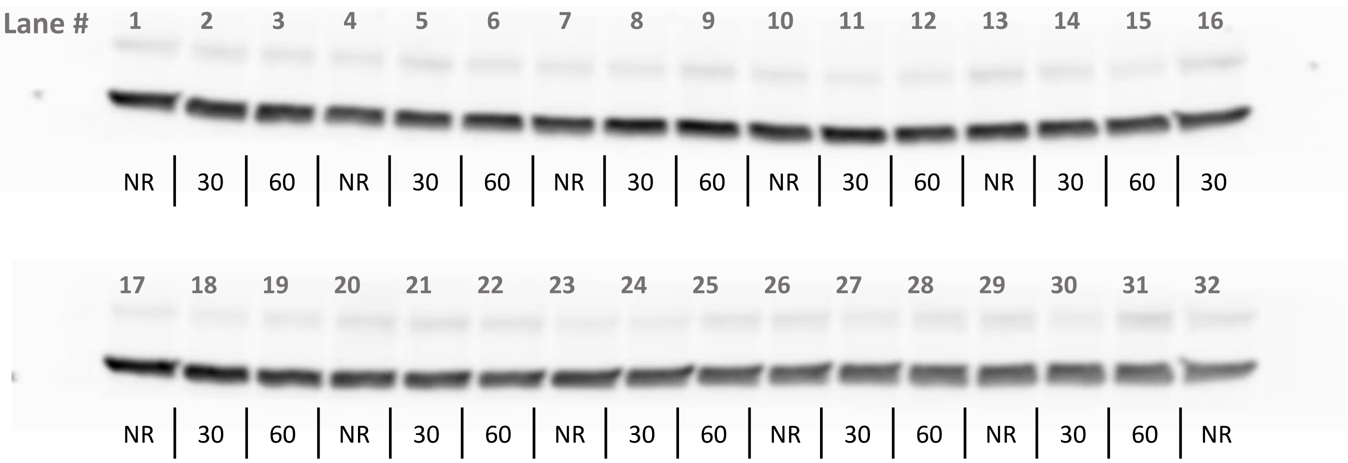

OFC

pAKT-Thr308 – 60 kDa (Figure S4)

Lane #	1	2	3	4	5	6	7	8	9	10	11	12	13	14	15	16

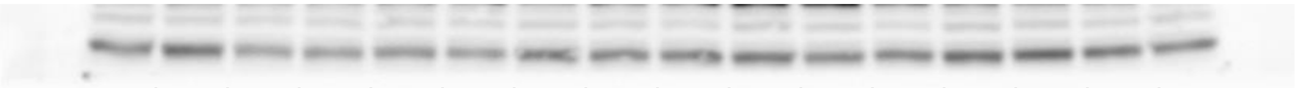

NR	30	60	NR	30	60	NR	30	60	NR	30	60	NR	30	60	30

17	18	19	20	21	22	23	24	25	26	27	28	29	30	31	32

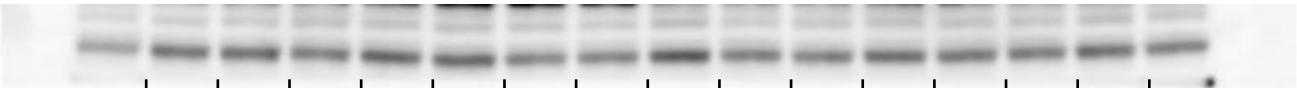

NR	30	60	NR	30	60	NR	30	60	NR	30	60	NR	30	60	NR

pAKT-Ser473 – 60 kDa (Figure S4)

Lane #	1	2	3	4	5	6	7	8	9	10	11	12	13	14	15	16

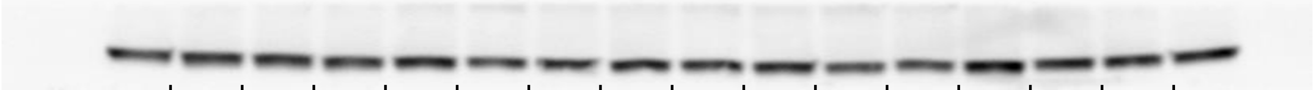

NR	30	60	NR	30	60	NR	30	60	NR	30	60	NR	30	60	30

17	18	19	20	21	22	23	24	25	26	27	28	29	30	31	32

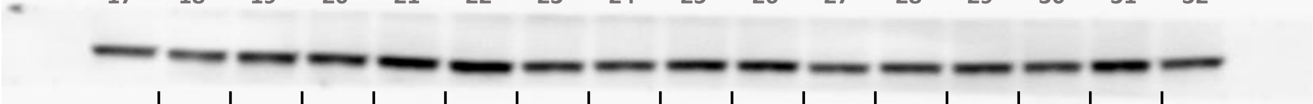

NR	30	60	NR	30 *	60	NR	30	60	NR	30	60	NR	30	60	NR

Total AKT - 60 kDa (Figure S4)

Lane #	1	2	3	4	5	6	7	8	9	10	11	12	13	14	15	16

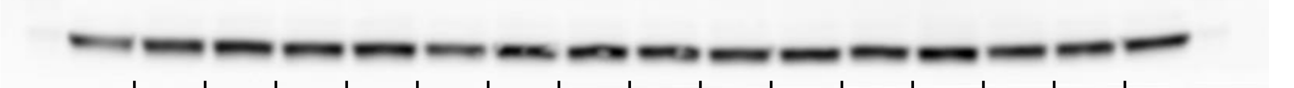

NR	30	60	NR	30	60	NR	30	60	NR	30	60	NR	30	60	30

17	18	19	20	21	22	23	24	25	26	27	28	29	30	31	32

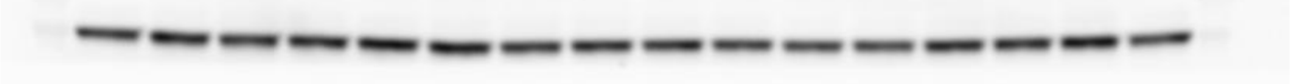

NR	30	60	NR	30	60	NR	30	60	NR	30	60	NR	30	60	NR

OFC

pERK1/2-Thr202/Tyr204 – 44/42 kDa (Figure S4)

Lane # 1 2 3 4 5 6 7 8 9 10 11 12 13 14 15 16

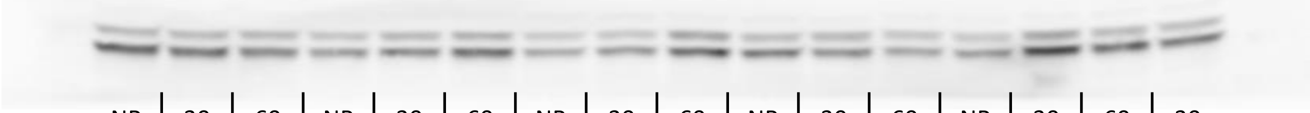

NR | 30 | 60 | NR | 30 | 60 | NR | 30 | 60 | NR | 30 | 60 | NR | 30 | 60 | 30

17 18 19 20 21 22 23 24 25 26 27 28 29 30 31 32

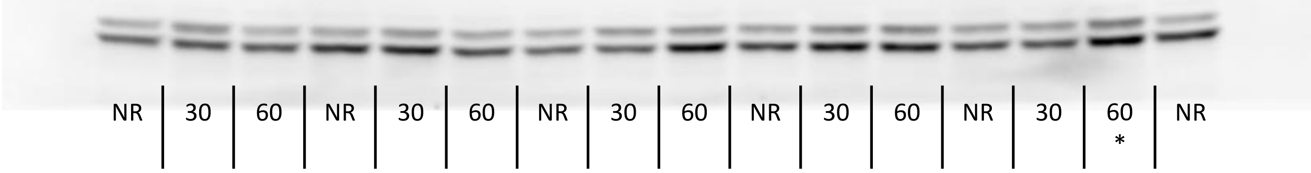

NR | 30 | 60 | NR | 30 | 60 | NR | 30 | 60 | NR | 30 | 60 | NR | 30 | 60 * | NR

Total ERK1/2 – 44/21 kDa (Figure S4)

Lane # 1 2 3 4 5 6 7 8 9 10 11 12 13 14 15 16

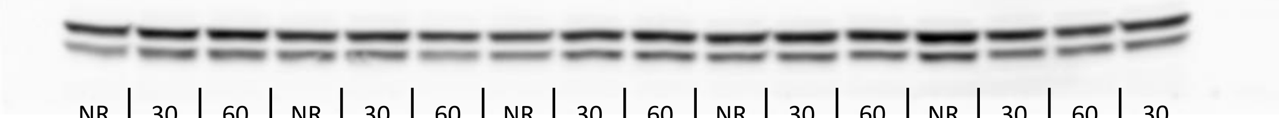

NR | 30 | 60 | NR | 30 | 60 | NR | 30 | 60 | NR | 30 | 60 | NR | 30 | 60 | 30

17 18 19 20 21 22 23 24 25 26 27 28 29 30 31 32

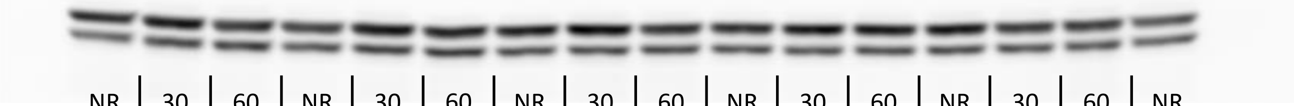

NR | 30 | 60 | NR | 30 | 60 | NR | 30 | 60 | NR | 30 | 60 | NR | 30 | 60 | NR

GAPDH – 37 kDa (Figure S4)

Lane # 1 2 3 4 5 6 7 8 9 10 11 12 13 14 15 16

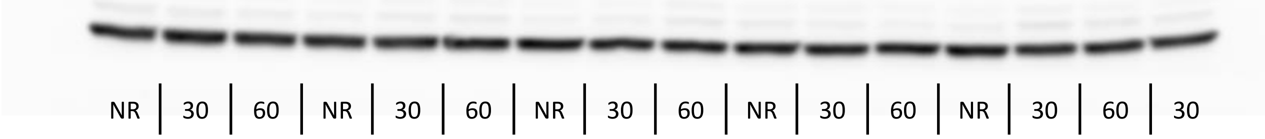

NR | 30 | 60 | NR | 30 | 60 | NR | 30 | 60 | NR | 30 | 60 | NR | 30 | 60 | 30

17 18 19 20 21 22 23 24 25 26 27 28 29 30 31 32

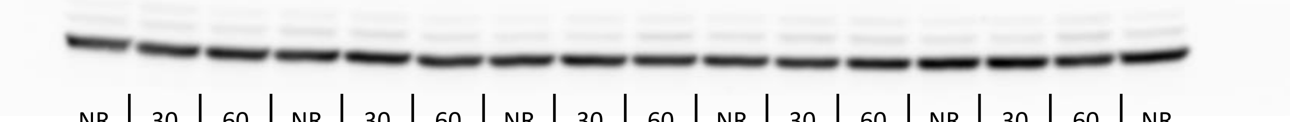

NR | 30 | 60 | NR | 30 | 60 | NR | 30 | 60 | NR | 30 | 60 | NR | 30 | 60 | NR

OFC

pGSK3β-Ser9 – 46 kDa (Figure S4)

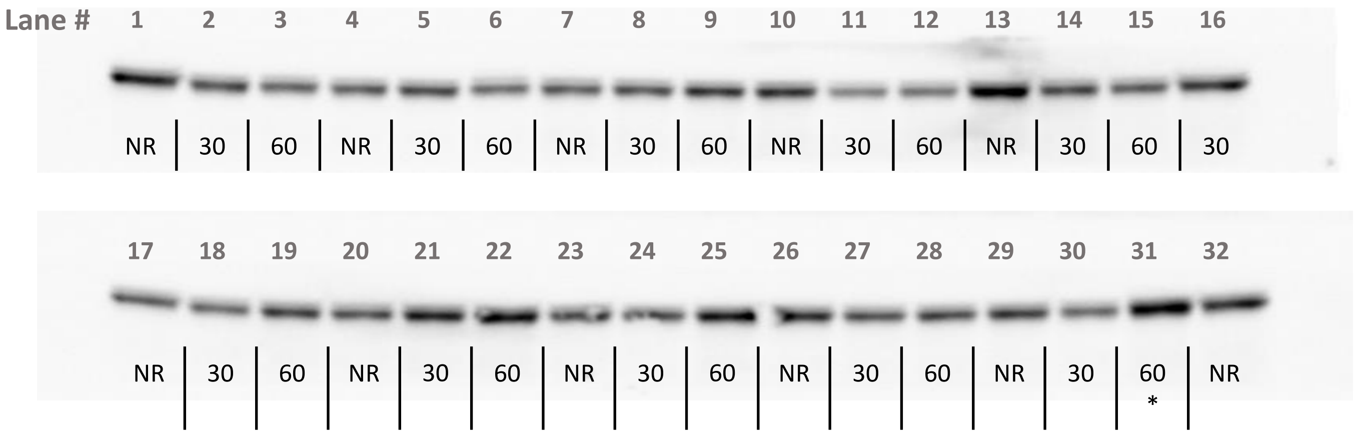

Total GSK3β – 46 kDa (Figure S4)

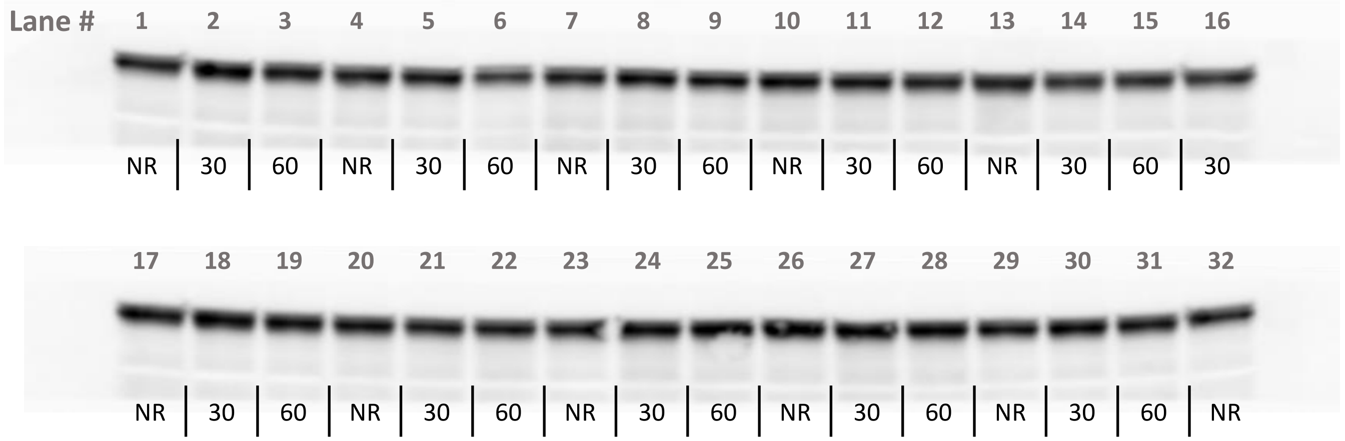

OFC

pCRMP2-Thr514 – 60-80 kDa (Figure S4)

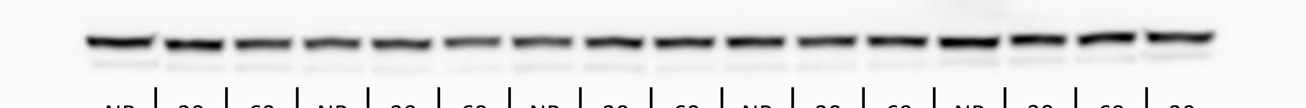

Lane #	1	2	3	4	5	6	7	8	9	10	11	12	13	14	15	16
	NR	30	60	NR	30	60	NR	30	60	NR	30	60	NR	30 *	60	30

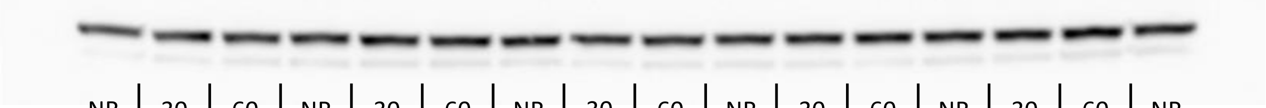

	17	18	19	20	21	22	23	24	25	26	27	28	29	30	31	32
	NR *	30	60	NR	30	60	NR	30 *	60	NR	30	60	NR	30	60	NR

Total CRMP2 – 60-80 kDa (Figure S4)

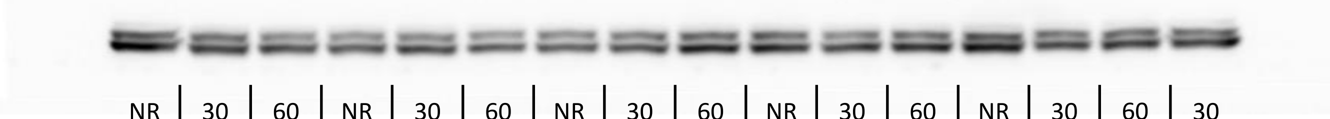

Lane #	1	2	3	4	5	6	7	8	9	10	11	12	13	14	15	16
	NR	30	60	NR	30	60	NR	30	60	NR	30	60	NR	30	60	30

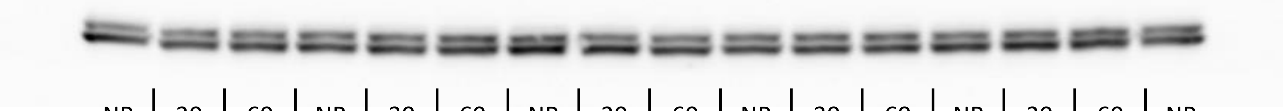

	17	18	19	20	21	22	23	24	25	26	27	28	29	30	31	32
	NR	30	60	NR	30	60	NR	30	60	NR	30	60	NR	30	60	NR

GAPDH – 37 kDa (Figure S4)

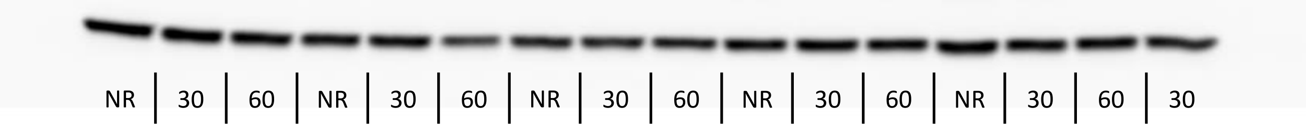

Lane #	1	2	3	4	5	6	7	8	9	10	11	12	13	14	15	16
	NR	30	60	NR	30	60	NR	30	60	NR	30	60	NR	30	60	30

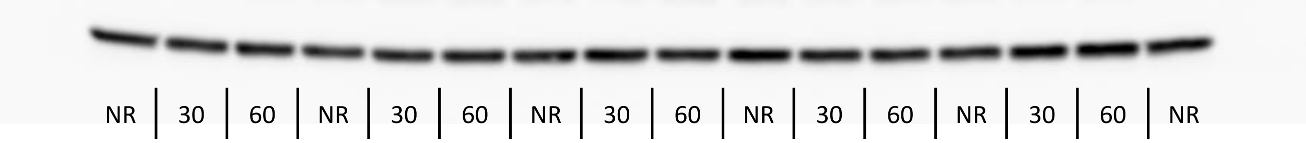

	17	18	19	20	21	22	23	24	25	26	27	28	29	30	31	32
	NR	30	60	NR	30	60	NR	30	60	NR	30	60	NR	30	60	NR

Experiment 4

Amygdala tissues were collected two hours after alcohol memory retrieval (R), or without a prior retrieval procedure (NR).

The membrane was cut prior to the blotting process, as illustrated below, therefore image of the complete membrane is not available.

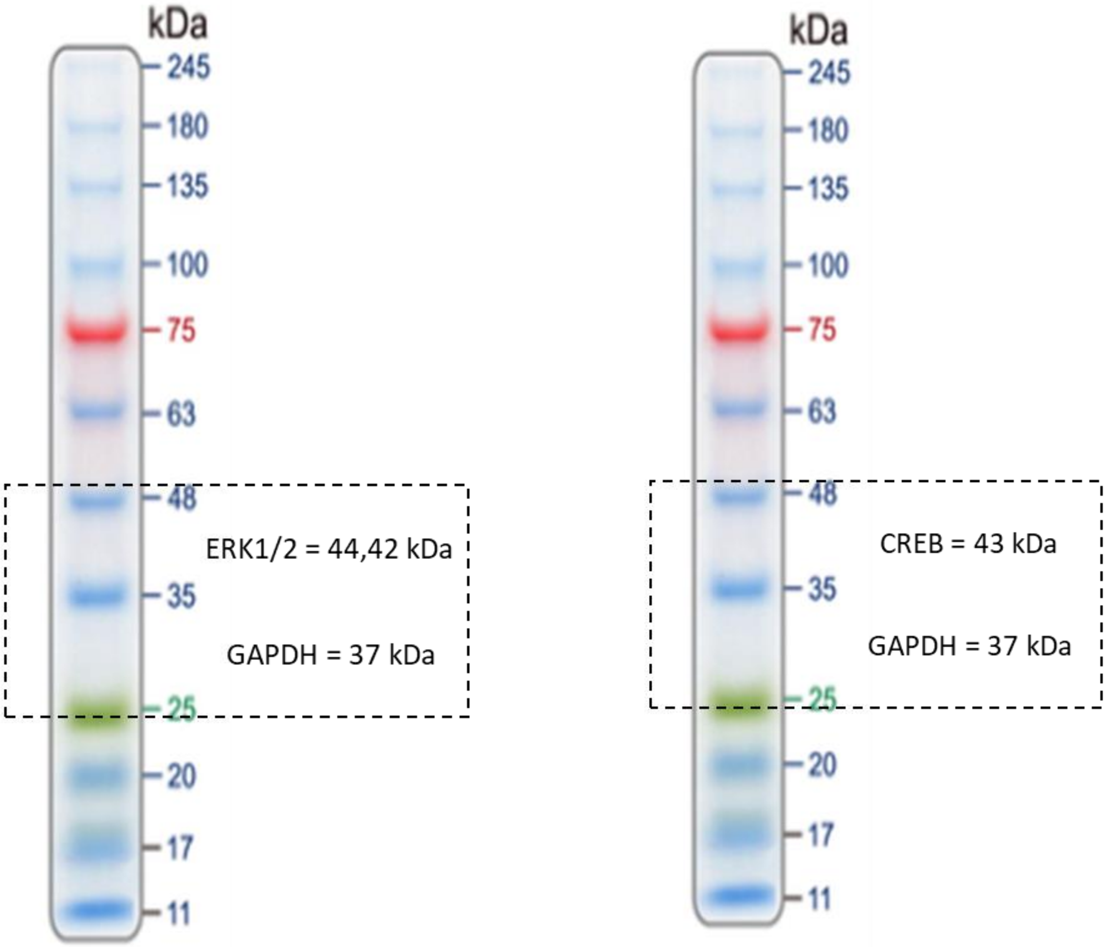

pERK1/2-Thr202/Tyr204 – 44/42 kDa (Figures 4C and S6)

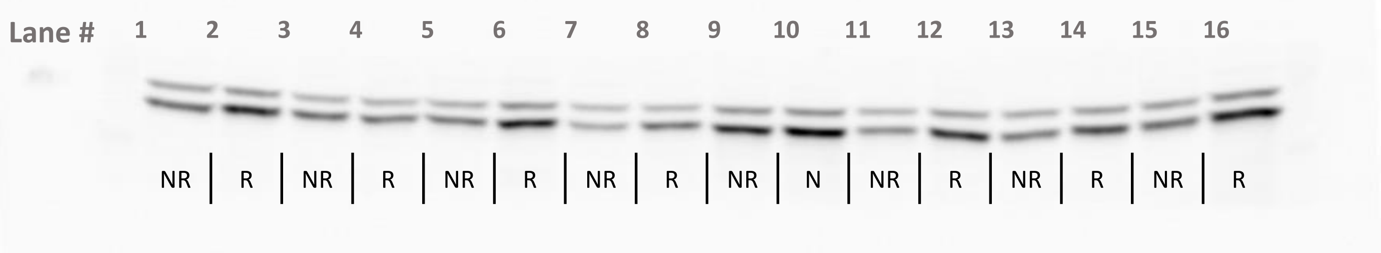

Total ERK1/2 – 44/21 kDa pERK1/2-Thr202/Tyr204 – 44/42 kDa (Figures 4C and S6)

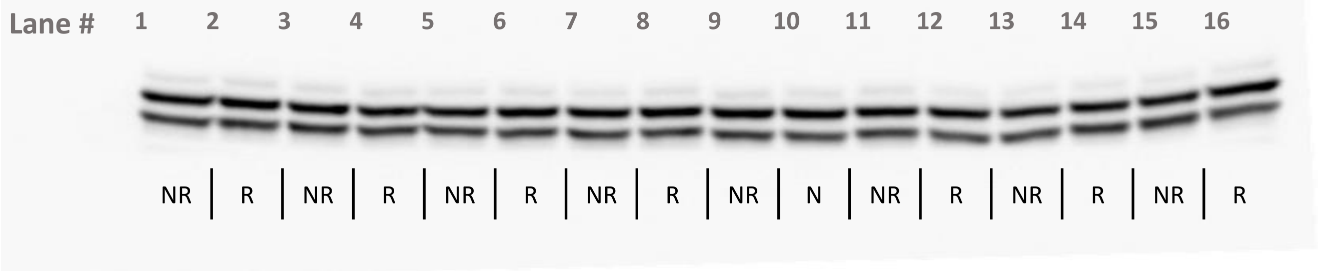

GAPDH – 37 kDa (Figure S6, not in the main figure)

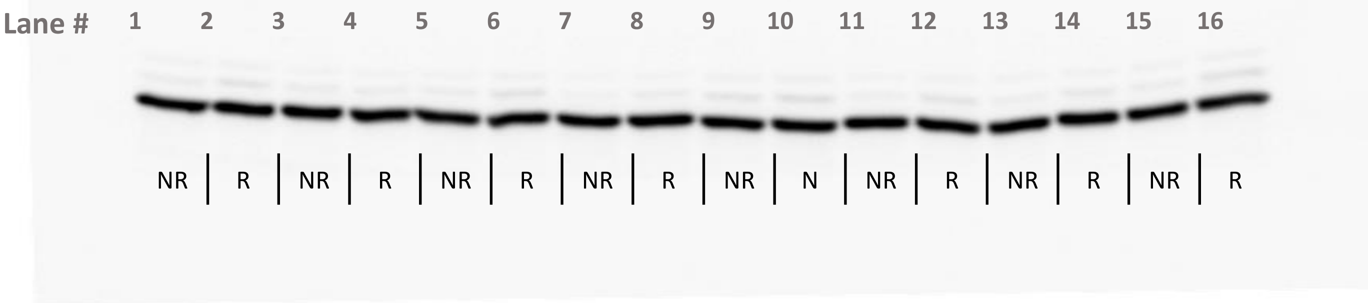

pCREB-Ser133 – 43 kDa (Figures 4C and S6)

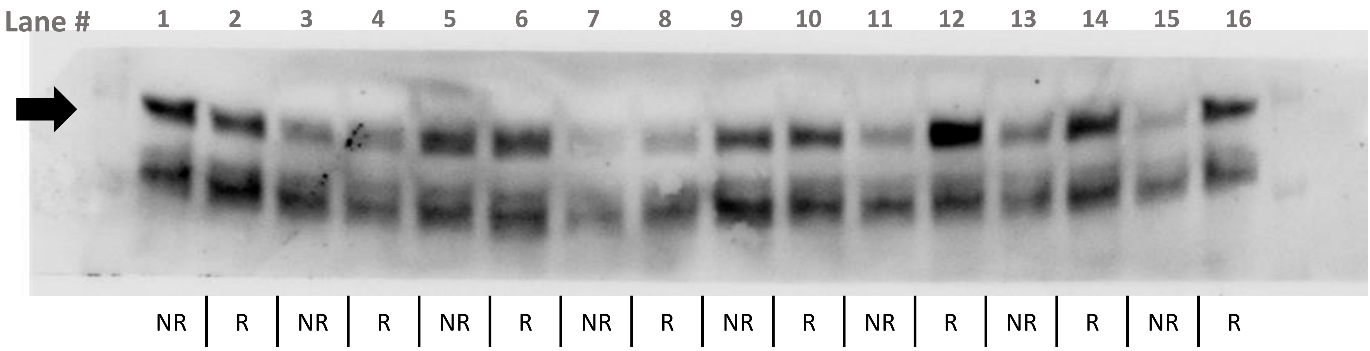

Total CREB-Ser133 – 43 kDa (Figures 4C and S6)

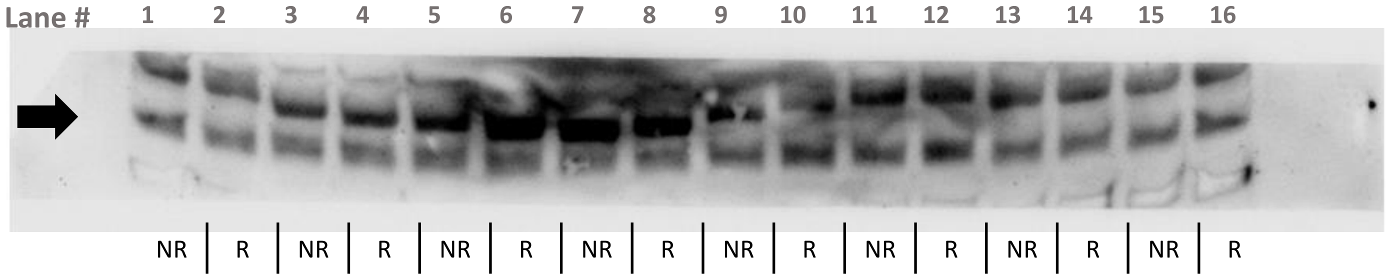

GAPDH – 37 kDa (Figure S6, not in the main figure)

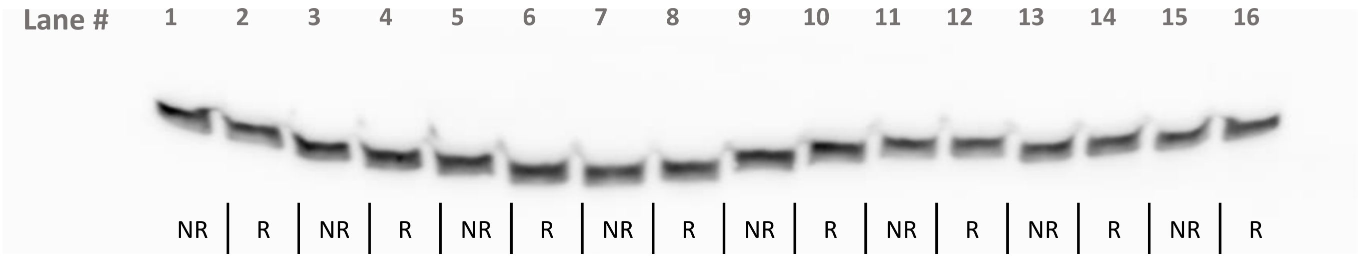

Experiment 5

Amygdala tissues were collected 90 min after injection of vehicle (V) or the MERK1/2 inhibitor SL-327 (SL).

The membrane was cut prior to the blotting process, as illustrated below, therefore image of the complete membrane is not available.

* Sample that was identified as an outlier to its experimental group (z>2) and was therefore removed from the analysis.

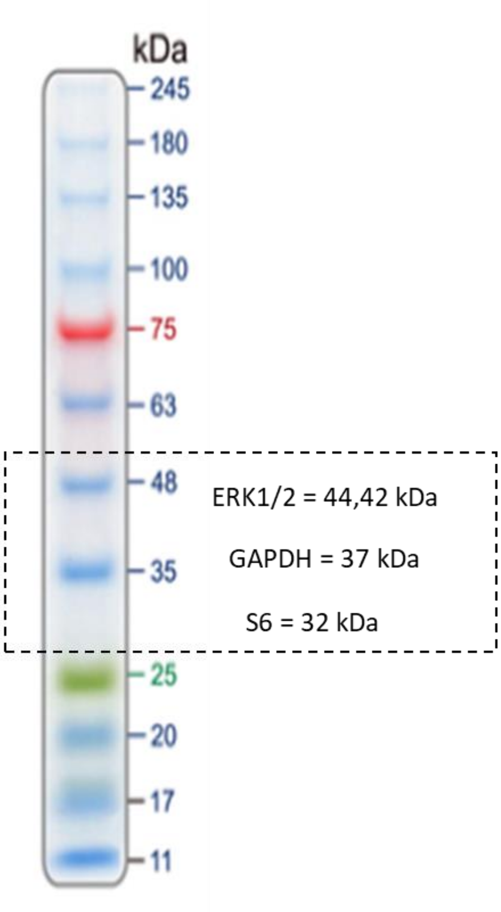

pERK1/2-Thr202/Tyr204 – 44/42 kDa (Figures 6 and S7)

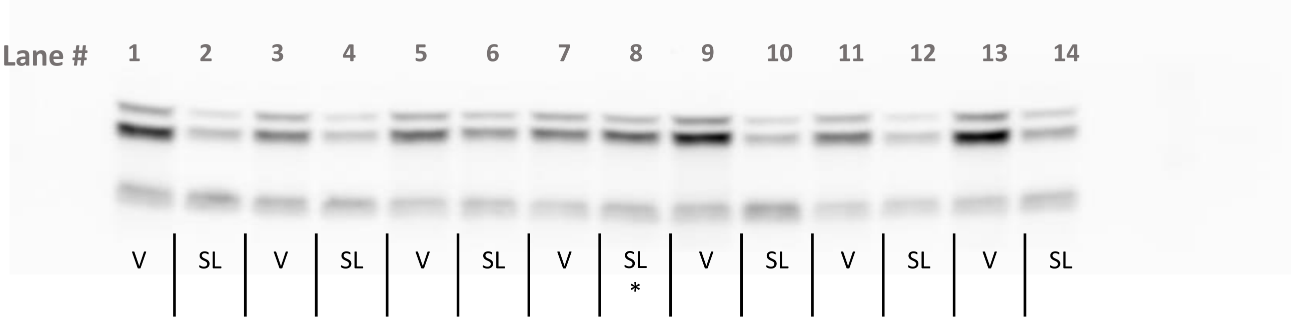

Total ERK1/2 – 44/21 kDa (Figures 6 and S7)

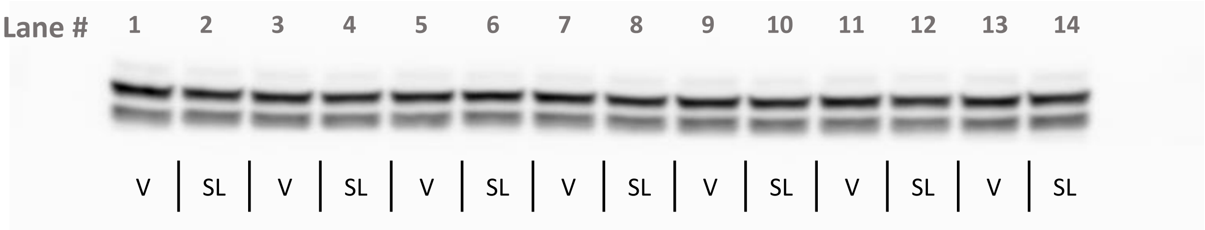

pS6 – 32 kDa (Figures 6 and S7)

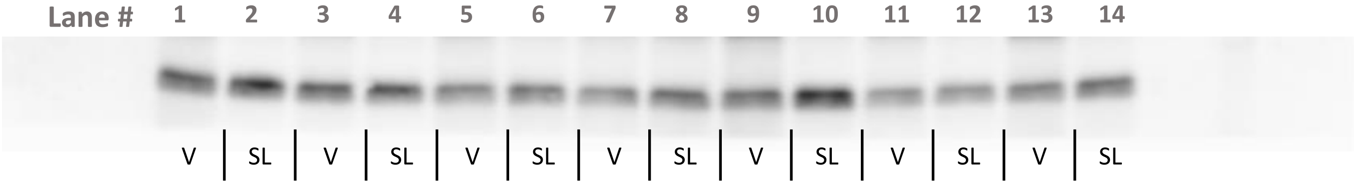

Total S6 – 32 kDa (Figures 6 and S7)

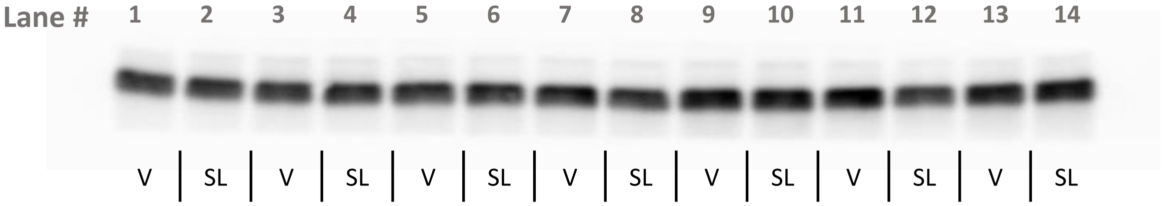

GAPDH – 37 kDa (Figure S7, not in the main figure)

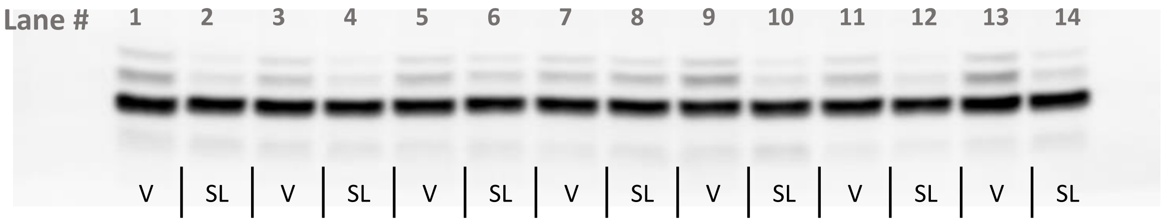

Supplement: Supplementary file 1 [file ijms-25-05478-s001.zip › ijms-2907258-supplementary.pdf]
